# Rice diversity panel provides accurate genomic predictions for complex traits in the progenies of biparental crosses involving members of the panel

**DOI:** 10.1007/s00122-017-3011-4

**Published:** 2017-11-14

**Authors:** M. Ben Hassen, T. V. Cao, J. Bartholomé, G. Orasen, C. Colombi, J. Rakotomalala, L. Razafinimpiasa, C. Bertone, C. Biselli, A. Volante, F. Desiderio, L. Jacquin, G. Valè, N. Ahmadi

**Affiliations:** 1 Department of Agriculture and Environmental Sciences, University of Milan, Via Giovanni Celoria, 2, 20133 Milan, Italy; 2 Cirad, UMR AGAP, Avenue Agropolis, 34398 Montpellier Cedex 5, France; 3 Fondazione Parco Tecnologico Padano, Via Einstein, Loc. Cascina Codazza, 26900 Lodi, Italy; 4 FOFIFA, Antananarivo, Madagascar; 5 CREA-Council for Agricultural Research and Economics, Research Center for Genomics and Bioinformatics, Via S. Protaso 302, 29017 Fiorenzuola d’Arda, PC Italy; 6 CREA-Council for Agricultural Research and Economics, Research Center for Cereal and Industrial Crops, S. S. 11 to Torino Km 2.5, 13100 Vercelli, Italy

## Abstract

**Key message:**

Rice breeding programs based on pedigree schemes can use a genomic model trained with data from their working collection to predict performances of progenies produced through rapid generation advancement.

**Abstract:**

So far, most potential applications of genomic prediction in plant improvement have been explored using cross validation approaches. This is the first empirical study to evaluate the accuracy of genomic prediction of the performances of progenies in a typical rice breeding program. Using a cross validation approach, we first analyzed the effects of marker selection and statistical methods on the accuracy of prediction of three different heritability traits in a reference population (RP) of 284 inbred accessions. Next, we investigated the size and the degree of relatedness with the progeny population (PP) of sub-sets of the RP that maximize the accuracy of prediction of phenotype across generations, i.e., for 97 F_5_–F_7_ lines derived from biparental crosses between 31 accessions of the RP. The extent of linkage disequilibrium was high (*r* ^2^ = 0.2 at 0.80 Mb in RP and at 1.1 Mb in PP). Consequently, average marker density above one per 22 kb did not improve the accuracy of predictions in the RP. The accuracy of progeny prediction varied greatly depending on the composition of the training set, the trait, LD and minor allele frequency. The highest accuracy achieved for each trait exceeded 0.50 and was only slightly below the accuracy achieved by cross validation in the RP. Our results thus show that relatively high accuracy (0.41–0.54) can be achieved using only a rather small share of the RP, most related to the PP, as the training set. The practical implications of these results for rice breeding programs are discussed.

**Electronic supplementary material:**

The online version of this article (10.1007/s00122-017-3011-4) contains supplementary material, which is available to authorized users.

## Introduction

Genomic selection (GS) arose from the conjunction of new high-throughput marker technologies and new statistical methods (Meuwissen et al. 2001). GS allows analysis of the genetic architecture for complex traits in the framework of infinitesimal model effects. It consists in (1) using all markers (often large numbers) simultaneously to build a model of genotype–phenotype relationships in a training population (TP), thus accounting also for linkage disequilibrium (LD) among markers, and (2) using the model to predict the genomic estimate of breeding values (GEBV) of candidates in a breeding population (CP) (Meuwissen et al. 2001; Jannink et al. 2010). The effectiveness of GS depends, among other factors, on the degree of correlation between the predicted GEBV and the true genetic value, i.e., the accuracy of prediction. In practice, the accuracy of prediction is evaluated by the correlation between GEBV and the realized phenotype.

Prospects for the applications of GS in plant breeding have given rise to many studies using simulations or experimental data. The effect of the statistical method on the accuracy of GEBV has been widely analyzed (Heslot et al. 2012). The general conclusion is that there is no single best statistical method and that the accuracy of the different methods depends on other factors, such as the characteristics of the target trait, the density and distribution of the markers, the size and the structure of the TP, and the degree of relatedness between TP and CP. The characteristics of the target trait reported to influence the accuracy of predictions include heritability, the number of QTLs, the distribution of their allelic effects and frequencies, and the relative magnitude of additive and non-additive genetic variance (Hayes et al. 2009a; Jannink et al. 2010; Howard et al. 2014; Burstin et al. 2015). Regarding marker density, empirical studies have confirmed the theoretical stance that marker density should be high enough to ensure strong linkage disequilibrium (LD) with at least one marker for each QTL (Lorenzana and Bernardo 2009; Lorenz et al. 2011; Heffner et al. 2011; Poland et al. 2012; Heslot et al. 2013). Another set of factors that strongly influence the accuracy of predictions includes the size of the TP, its structure, and its relatedness with the CP. Accounting for population structure through stratified sampling in the TP can significantly improve the accuracy of the predictions (Albrecht et al. 2011; Grenier et al. 2015; Isidro et al. 2015). Methods have been developed to optimize the composition of the TP (Rincent et al. 2012; Akdemir et al. 2015), by maximizing the expected reliabilities for a given set of individuals.

GS issues that need more thorough empirical studies include the evaluation of accuracy of genomic prediction for making selection decisions in pedigree breeding within the progeny of biparental crosses (Desta and Ortiz 2014). Beyene et al. (2015) compared the genetic gain for grain yield realized in eight biparental crosses of maize under GS associated with rapid cycling, with conventional pedigree breeding and reported that the average genetic gain per year under GS was three times higher than that achieved by conventional breeding. However, such a GS breeding approach required separate model training for each biparental cross and phenotyping of the first generation of progeny, which increase the costs and duration of breeding cycles. A second approach, recently investigated in a number of crops, is the use of a reference set to train the prediction model and the use of this model to predict the performance of progenies from biparental crosses between members of the panel. For instance, Hofheinz et al. (2012) used a reference set of 310 inbred sugar beet lines to predict the test cross value of 56 inbred progeny derived from eight crosses between six lines of the reference set, and reported average prediction accuracy of 0.79 for sugar content. Sallam et al. (2015) used a training set of 168 barley lines and five sets of 96 progeny lines, representative of the breeding lines developed in five consecutive years (the training set included the parents of the progeny sets) and reported a prediction accuracy of around 0.50 for grain yield. Likewise, Gezan et al. (2017) used a panel representative of the Florida University strawberry breeding program and sets of progenies derived from the circular mating of 31 members of the panel and reported a prediction accuracy ranging from 0.16 to 0.77 depending on the traits and model fitting method used. However, this approach faces the challenge of differences in LD and allele frequencies between the reference panel and the progenies of individual biparental crosses, requiring thorough management of marker density. It also raises the question of the choice of prediction model to capture either markers’ LD with QTLs or the marker-based relationship between the training set and the progenies sets, both of which require knowledge of the genetic architecture of the target traits (Zhong et al. 2009; Zhang et al. 2017).

Rice (*Oryza sativa*) is the world’s most important staple food and will continue to be so in the coming decades. Genetic improvement is one of the major pillars of sustainable adaptation of rice production to ongoing global changes (Atlin et al. 2017). GS is expected to accelerate genetic gain for traits such as yield potential and adaptation to constraints related to climate change and the efficient use of resources (water, nitrogen, etc.) (Ashikari 2017; Atlin et al. 2017). So far, however, GS studies on rice have mainly explored the cross validation approach within diversity panels (Table 1).

**Table 1:** Genomic selection studies conducted on rice

Plant materiel	Phenotypic data	Genotypic data	Statistical methods	Range of accuracy of GEBV	Main conclusion	References
110 Asian cultivars	Eight traits including days to flowering (FL)	3071 SNPs	rrBLUP, ENet, GBLUP, RKHS, RF, Lasso, BL, EBL, wBSR	FL: 0.65–0.85	Reliability depended to a great extent on the traits targeted. Reliability was low when only a small number of cultivars were used for validation	Onogi et al. (2014)
Highly structured diversity panel of 413 accessions	Eight traits including grain yield (GY), flowering date (FL) and plant height (PH)	36,901 SNPs (1 SNP per 10 Kb)	GBLUP	FL: 0.25–0.60 PH: 0.25–0.55 GY: 0.20–0.50	Maximizing the phenotypic variance captured by the training set is important for optimal performance. Stratified sampling of the training set ensures better accuracy than sampling based on the CDmean	Isidro et al. (2015)
	15 traits of rather high heritability, including flowering time (FL), plant height (PH) and protein content	36,901 SNPs (1 SNP per 10 Kb)	GBLUP, GBLUP-CPS	FL: 0.44–0.66 PH: 0.50–0.75	Prediction accuracy was affected by the genomic relationship between TP and VP and by genomic heritability in the TP and VP	Guo et al. (2014)
369 elite breeding lines	Six traits including days to flowering (FL) and grain yield (GY)	73,147 SNPs	rrBLUP, BL, RKHS, RF,	FL: 0.35–0.65 PH: 0.15–0.35 GY: 0.10–0.30	Using one marker every 0.2 cM was sufficient for genomic selection in this collection of rice breeding material. rrBLUP was the most efficient statistical method for GY where no marked effect of QTLs was detected by GWAS	Spindel et al. (2015)
354 S3: 4 lines	Days to flowering (FL), plant height (PH) and grain yield (GY)	8336 SNPs 1 marker per 44.8 kb	rrBLUP, GBLUP, Lasso, BL	FL: 0.20–0.30 PH: 0.50–0.60 GY: 0.20 –0.31	Accuracy of GEBV was affected by (i) relatedness between TP and CP, (ii) trait heritability and interaction between traits and all the other factors studied (prediction models, LD, MAF, composition of the TP)	Grenier et al. (2015)
115 lines of hybrids	Eight traits including grain yield (GY), and plant height (PH)	2395,866 SNPs	GBLUP, GBLUP dominance effects	FL: – PH: 0.45–0.86 GY: 0.13–0.34	Model including the dominance effect provided more accurate prediction, particularly in multi-traits scenario for a low-heritability target trait, with highly correlated auxiliary traits	Wang et al. (2017)

Here we report the first empirical study that assesses the accuracy of genomic prediction among the progeny of rice biparental crosses using a reference panel for model training. It was undertaken in the framework of a rice breeding program conducted according to the most common rice breeding scheme, i.e., pedigree breeding within the progenies of biparental crosses, the parents being chosen within a working collection of inbred accessions. The reference panel was composed of 284 inbred accessions of the breeding program’s working collection. The progeny population was composed of 97 inbred lines derived from 36 biparental crosses between 31 accessions of the working collection. The main objectives of this study were to investigate (1) the size and the degree of relatedness of the reference panel that maximize the accuracy of prediction of phenotype of progeny lines from several biparental crosses; (2) the effects of marker selection and statistical methods on prediction accuracy across generations, for three traits with different heritability; and (3) the accuracy of genomic prediction among the progeny of individual biparental crosses.

## Materials and methods

### Plant material

The plant material comprised a diversity panel of 284 accessions and 97 advanced (F_5_–F_7_) inbred lines. The diversity panel represents the working collection of the rice breeding program of Research Centre for Cereal and Industrial Crops (CREA), Vercelli, Italy. It is composed of 139 accessions of Italian origin and 145 accessions of diverse geographic origin (Supplementary Table 1), all belonging to the *japonica* subspecies of *O. sativa* and all adapted to cultivation in the irrigated rice ecosystem of temperate Mediterranean Europe (Faivre-Rampant et al. 2011; Biscarini et al. 2016). Hereafter, this diversity panel is referred to as the “reference population” (RP). The 97 advanced lines were derived from 36 biparental crosses (including five backcrosses) involving 31 accessions of the diversity panel (Supplementary Table 1). The number of progenies per cross ranged from 1 to 20 (Supplementary Table 2; Supplementary Fig. 1). In the present study, these 97 lines constituted the “progeny population” (PP).

### Field trials and phenotyping

Phenotyping of RP and PP took place at the CREA experimental station (45°19′24.00″N; 8°22′26.28″E; 134 m asl.), in an irrigated cropping system with standard crop management. The RP was phenotyped during the 2012 and 2013 rice cropping seasons under a complete randomization experimental design with three replicates per accession. The size of the individual plot was 1.70 m × 0.40 m, each plot contained three rows of 60 seeds. The PP was phenotyped during the 2014 and 2015 rice cropping seasons under randomized complete block design with three replicates. The size of the individual plot was 1.20 m × 0.80 m, each plot contained six rows of 40 seeds.

The target traits for both RP and PP were days to flowering (FL), panicle weight (PW), and the nitrogen balance index (NI). FL was recorded as the number of days after sowing, when 50% of the plants in the plot were in flower. In the experiments related to RP, PW (g) was recorded by weighing a random sample of 50 panicles in the plot, and, in the experiments related to PP, by weighing 100 representative panicles. The measurements were harmonized to represent the weight of 100 panicles. NI, an indicator of the plant nitrogen status (Tremblay et al. 2012), was recorded using a Dualex™ instrument (Goulas et al. 2004), 7 to 10 days after the flowering date, a period during which the nitrogen status of the plant is stable. In each plot, three measurements were made on the adaxial and the abaxial sides of a flag leaf on three plants. The 18 measurements were then averaged to obtain a plot level NI.

### Analysis of phenotypic data

Phenotypic data for each trait and each population were analyzed separately using the proc mixed procedure of SAS 9.2 (SAS Institute, Cary NC, USA). The mixed model used for the RP was:

$$Y_{ijk} = \mu + g_{i} + y_{j} + \left( {gy} \right)_{ij} + e_{ijk}\quad \left( {\text{RP model}} \right),$$

where $Y_{ijk}$ is the observed phenotype of genotype *i* in year *j* and in plot *k*, $\mu$, the overall mean, $g_{i}$, the genotype effect, $y_{j}$, the year effect, $\left( {gy} \right)_{ij}$, the interaction between genotype *i* and year *j*, and $e_{ijk}$ the residual. Except for the overall mean, all the effects were considered random and the variance components were tested using the Wald Z-statistic tests, which are appropriate for large sample sizes.

The mixed model used for the PP was:

$$Y_{ijk} = \mu + g_{i} + y_{j} + r\left( y \right)_{jk} + \left( {gy} \right)_{ij} + e_{ijk}\quad \left( {\text{PP model}} \right),$$

where $Y_{ijk}$, $\mu$, $g_{i}$, $y_{j}$, $\left( {gy} \right)_{ij}$ and $e_{ijk}$ have the same meaning as in the RP model, and $r\left( y \right)_{jk}$, is the replicate within year effect. As in the previous model, all the effects except $\mu$ were considered random.

A model-based diagnostic analysis was run for each field trial and each trait within the mixed model framework above, to detect potential outliers among the individual data points (plot level). The restricted likelihood distance (RLD) output of the diagnostic procedure was used to identify outliers. RLD is a global measure of the influence of the observations jointly on all parameters. If $\varphi$ denotes the collection of all parameters in the model, i.e., including fixed ($\beta$) and random ($\theta$), then $RLD\left( U \right) = 2\left\{ {lR\left( {\hat{\varphi }} \right) - lR\left( {\hat{\varphi }\left( U \right)} \right)} \right\}$ is twice the difference between the restricted log-likelihood evaluated at the full-data estimates $\hat{\varphi }$ and at the reduced-data estimates $\hat{\varphi }$ (U). The distribution of the RLD values of the accessions was inspected visually and when one was considered too high, the corresponding plot data were compared, and outlier plots were either corrected or discarded. This procedure resulted in the elimination of five data points (of PW) in the 2013 field trial involving the RP. The eliminated data were considered as missing in the following steps of data analysis.

In addition to the above-mentioned diagnostic analysis, spatial homogeneity of the experimental fields, where RP phenotyping took place under a complete randomization design, was surveyed by visual analysis of the heat-map of the residuals (Supplementary Fig. 2). A slight discrepancy in the random distribution of the residuals was observed for NI only.

Broad sense heritability of accession means, *H* ^2^, was calculated for each trait in each population using the formula of Holland et al. (2003) as follows:

$$H^{2} = \frac{{\sigma_{g}^{2} }}{{\sigma_{g}^{2} + \frac{{\sigma_{gy}^{2} }}{ny} + \frac{{\sigma_{e}^{2} }}{nr}}}$$

where *ny* represents the mean number of years in which the accessions were tested and *nr*, the mean number of plots per accession across years. The means were calculated as harmonic means. Finally, adjusted means of accessions ($\hat{Y}_{i} = \hat{\mu } + \hat{g}_{i}$, with *g*, as random effect) were extracted for each trait to be used as phenotypes in the genomic prediction models.

### Genotyping and genotypic data

The genotyping procedure is detailed in Biscarini et al. (2016). Briefly, genomic DNA was isolated from 3-week-old leaves using the DNeasy Plant Mini Kit (QIAGEN, Milan, Italy) with a TECAN Freedom EVO150 liquid handling robot (TECAN Group Ltd, Männedorf, Switzerland). DNA digestion was performed using ApeKI restriction enzyme. Digested DNAs were ligated to 12 of 0.6/adapter pairs (optimized to guarantee good quality libraries in rice), and the 96-plex library constructed according to the genotyping by sequencing (GBS) protocol. The libraries were loaded into a Genome Analyzer II (Illumina, Inc., San Diego, USA) for sequencing. The *Tassel* GBS pipeline v3.0 (Glaubitz et al. 2014) was used to filter the raw data, sequence alignment to the rice reference genome (*Os*-*Nipponbare*-*Reference*-*IRGSP*-*1.0*), and for SNP calling. The procedure yielded 246,554 SNPs with a call rate ≥ 80%. Filtering of the matrix for missing data with a threshold of 20% led to 70,530 SNPs with an average rate of 9.2% missing data. Missing SNP genotypes were then imputed using the FILLIN (Fast, Inbred Line Library ImputatioN) algorithm in the *Tassel* GBS pipeline v3.0, with default settings. Filtering of this matrix for the rate of heterozygoty (threshold of 5%) and for a minor allele frequency (MAF, threshold of 2.5%) among the RP accessions and PP lines, considered together, led to a final working set of 43,686 SNP loci. The genotypic data are available at http://tropgenedb.cirad.fr/tropgene/JSP/interface.jsp?module=RICE, (Choose Tab Studies) as GS-Ruse_CREA_GBSgenotype_RP&PP.

### Genotypic characterization of RP and PP

The genetic structure of the two populations was analyzed jointly using a distance-based method. First, a matrix of 4824 SNPs was extracted from the working genotypic dataset of 43,686 SNPs, by discarding loci that had imputed data and by imposing a minimum distance of 10 kb between two adjacent loci. Then an unweighted neighbor-joining tree based on a simple matching matrix was constructed using DarWin v6 (Perrier and Jacquemoud-Collet 2006).

Pairwise LD between SNP loci was calculated separately in RP and PP at the level of the individual chromosome, using the working genotypic dataset of 43,686 SNPs and the *r* ^2^ estimator proposed by Rogers and Huff (2009) for non-phased genotypic data.

### Genomic prediction methods

Three statistical methods were tested: genomic best linear unbiased prediction (GBLUP), reproducing kernel Hilbert spaces regressions (RKHS) and BayesB (Meuwissen et al. 2001). The GBLUP method (VanRaden 2008) was implemented using the Expectation–Maximization convergence algorithm and the genomic matrix *G* = *XX*′, *X* being the centered genotype matrix containing values of − 1, 0 and 1, with *N* × *P* dimension, where *N* is the number of entries and *P* the number of markers. For the RKHS regression (Gianola and van Kaam 2008), the Gaussian kernel $K\left( {x_{i} ,x_{j} } \right) = { \exp }\left( { - h ||x_{i} - x_{j} ||^{2} } \right)$ was used to build the kernel matrix (or the Gram matrix) between the marker genotype vectors $x_{i}$ and $x_{j}$, where $\left( {i,j} \right) \in \left\{ {1, \ldots ,N} \right\}^{2}$. The rate of decay parameter *h*, also known as the bandwidth parameter, was estimated using the *k*-folds cross validation method implemented in the Tune_kernel_Ridge_MM function of the R package *KRMM*. The R package *kernlab* (Karatzoglou et al. 2004) was used to compute the kernel matrix. Both GBLUP and RKHS methods were implemented using the *KRMM* package (https://cran.r-project.org/web/packages/KRMM/index.html) described by Jacquin et al. (2016). For BayesB, the model that specified two component mixtures prior with a point of mass at zero and a scaled-*t* slab for marker effect (Meuwissen et al. 2001) was implemented using the *BGLR* statistical package (Pérez and de los Campos 2014). The default parameters for prior specification were used and the number of iterations for the Markov chain Monte Carlo (MCMC) algorithm was set to 12,000 with a burn-in period of 2000.

### Construction of the incidence matrices

Twenty-one incidence matrices were constructed to investigate the effect of LD (7 threshold levels) and MAF (3 threshold levels), on the accuracy of genomic predictions within the RP. The seven LD thresholds, *r* ^2^ ≤ 0.25, ≤ 0.36, ≤ 0.49, ≤ 0.64, ≤ 0.81, ≤ 0.98 and ≤ 1, were chosen so as to correspond to the square of seven thresholds of Pearson correlation between genotype at each pair of loci of a given chromosome (− 0.5 ≤ *r* ≤ + 0.5, − 0.6 ≤ *r* ≤ + 0.6, − 0.7 ≤  *r*  ≤ +0.7, − 0.8 ≤ *r* ≤ + 0.8, − 0.9 ≤ *r* ≤ + 0.9, − 0.99 ≤ *r* ≤ + 0.99, − 1 ≤ *r* ≤ + 1). The choice of the three MAF thresholds (≥ 5, ≥ 10, and ≥ 20%) was intended to represent its distribution (first quartile, median and third quartile) within RP and PP.

The incidence matrices were constructed as follows: (1) using the genotypic dataset (*N* = 289 entries and *P* = 43,686 SNPs), markers were selected based on the three MAF thresholds (≥ 5, ≥ 10, and ≥ 20%); (2) for each of the three resulting matrices, the pairwise LD between markers was calculated for each chromosome; (3) for each marker and for each LD threshold, redundancy information was computed as the number of times the pairwise LD with other markers was above the LD threshold; (4) markers with a redundancy level above an empirical threshold of 30 were discarded. This empirical threshold of redundancy represented a good compromise between our two objectives, one to reduce redundancy, which varied widely between SNPs, the other to dispose of an ample range of marker density among the 21 incidence matrices. As a result, marker density ranged from 8.7 to 83.5 SNP per Mb for the seven LD thresholds under MAF ≥ 5%, from 5.0 to 69.9 under MAF ≥ 10% and from 3.1 to 52.4 under MAF ≥ 20% (Table 2).

**Table 2:** Size of the incidence matrices used in the cross validation experiments in the reference population

LD (*r* ^2^)	Minor allele frequency (MAF)					
	≥ 5%		≥ 10%		≥ 20%	
	*N*	*D*	*N*	*D*	*N*	*D*
≤ 0.25	3322	8.7	1927	5.0	1173	3.1
≤ 0.36	5365	14.0	3450	9.0	2270	5.9
≤ 0.49	8324	21.7	5738	14.9	4013	10.5
≤ 0.64	12,099	31.5	8744	22.8	6095	15.9
≤ 0.81	16,923	44.1	12,652	34.2	8917	23.2
≤ 0.98	28,164	73.3	23,119	60.2	16,750	43.6
≤ 1	32,066	83.5	26,845	69.9	20,104	52.4

*N* total number of SNPs, *D* SNP density per Mb

### Cross validation experiments

The cross validation experiments used 189 (2/3) of the 284 accessions of the RP as the training set and the remaining 95 (1/3) accessions as the validation set. Each cross validation experiment was repeated 100 times using 100 independent partitioning of the accessions into the training set and validation set. For each independent partitioning, the correlation between the predicted and the observed phenotype was calculated, so as to obtain 100 correlations for each cross validation experiment. The accuracy of each cross validation experiment was computed as the mean value of the 100 correlations.

A total of 189 cross validation experiments were undertaken combining the above-described seven LD threshold levels, three MAF threshold levels, three prediction methods, and the three phenotypic traits. The same 100 independent partitioning of the training and validation sets was used for all 189 cross validation experiments.

### Genomic prediction across generations

Six scenarios, representing different degrees of relatedness between the training set and the progeny set and different sizes of the training set, were considered (Table 3). To this end, first, using pairwise Euclidian distances between each parental line and other accessions of the RP, the three closest accessions to each of the 31 parental accessions were identified. These accessions were then pooled to form the most related subset. Pooling led to a total of 58 accessions, because the closest accessions for some parents also happened to be the closest for other parents. Finally, this subset was combined, or not, with the parental lines and with the other accessions of RP to constitute the six training sets of the six prediction scenarios. For each scenario, the correlation between the predicted and the observed phenotypes of the 97 progeny lines was calculated, and represents the accuracy of the prediction experiment. In the case of scenario S6, in which the 31 accessions of the training set were randomly sampled 100 times from the RP excluding the parents, prediction accuracy was computed as the mean value of the 100 correlations between the predicted and the actual phenotypes of the 97 progeny lines. The objective of this random sampling was to reduce the risk of over-/under-estimation of prediction accuracy in this scenario. Comparisons between scenarios were consequently based on progeny prediction accuracy (PPA) data for the non-replicated prediction experiments, and on the average PPA for the replicated experiments in scenario S6.

**Table 3:** Scenarios for genomic prediction across generations

Scenario	Training set	Validation set
S1	31 parents	97 progeny
S2	58 related accessions	97 progeny
S3	31 parents + 58 related accessions	97 progeny
S4	31 parents + 252 accessions	97 progeny
S5	252 accessions, excluding the parents	97 progeny
S6	100 random sampling of 31 accessions, excluding the parents	97 progeny

The six scenarios were implemented with seven incidence matrices corresponding to the seven thresholds of LD used in the cross validation experiments, a unique MAF threshold of ≥ 5%, and three prediction methods (GBLUP, RKHS and BayesB). The accuracy observed under scenarios S1, S2 and S3 was also compared with the accuracy obtained by three training sets of equivalent sizes selected using the dedicated CDmean optimization method (Rincent et al. 2012).

To explore the accuracy of progeny prediction within the progenies of individual crosses, the correlation between the predicted and the observed phenotypes of the progeny lines of two crosses represented by a reasonably high number of advanced lines (Eurosis × Handao-11 and Giano × Vialone-Nano, represented by 20 lines and 9 lines, respectively) were also computed separately.

### Analysis of sources of variation in the accuracy of genomic prediction

The accuracy data (*r*) of all prediction experiments were transformed into a *Z*-statistic using the equation: $Z = 0.5 \left\{ {ln\left[ {1 + r\left] { - ln} \right[1 - r} \right]} \right\}$ and analyzed as a dependent variable in an analysis of variance. After estimation of confidence limits and means for Z, these were transformed back to *r* variable. For each trait, a separate ANOVA was performed for the correlations of all the PPA and of the average PPA in the progeny prediction experiments. In each case, ANOVA was performed to partition the variance of accuracy into different sources, with all effects declared as fixed, and following two models. The first model compared the effects of LD, MAF and prediction method in the cross validation experiments, and the effect of LD, scenario and prediction method in the progeny prediction experiments, with no interaction. The second model accounted for all the principal effects as well as for all possible first-order interactions.

## Results

### Phenotypic diversity of the three traits investigated

**Fig. 1:**
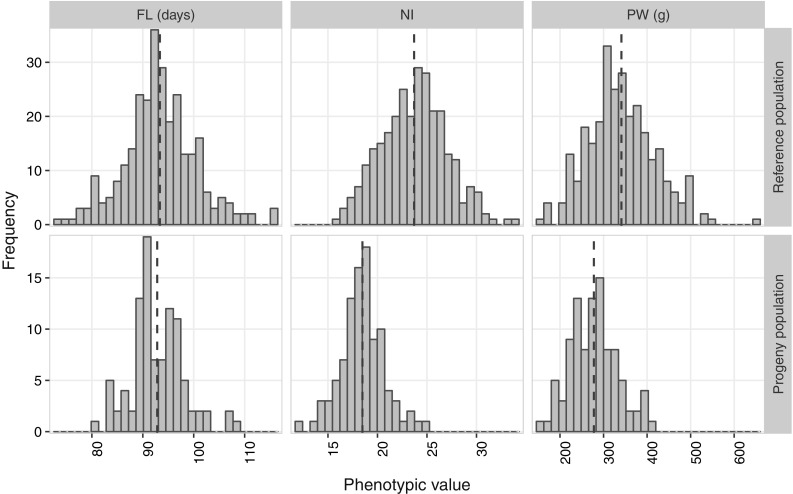
Distribution of phenotypic values for days to flowering (FL), nitrogen balance index (NI) and panicles weight (PW) in the reference and the progeny populations

The three traits investigated in the RP and PP populations exhibited a Gaussian distribution (Fig. 1). For all three traits, the extent of phenotypic diversity was broader in the RP than in the PP. Moreover, the distribution of NI and PW in the PP remained among the lowest values for these traits, leading to lower mean values. The narrower phenotypic diversity of PP is probably linked to its narrower genetic diversity (see below).

Separate ANOVA conducted in the RP and in the PP revealed a very highly significant effect of entry or genotype for the three traits (Table 4). The year effect was not significant, whereas the effects of genotype by year interaction were significant.

**Table 4:** Variance components of three phenotypic traits in the reference and progeny populations

Population	Factors	FL	NI	PW
Reference population	Genotype	47.78***	6.17***	5023.13***
	Year	16.82NS	2.96NS	222.18NS
	Year × genotype	4.36***	4.08***	889.8***
	Residual	5.95	16.74	2378.04
	*H* ^2^ (SE)	0.937 (0.007)	0.558 (0.052)	0.852 (0.018)
Progeny population	Genotype	23.2***	4.12***	2698.61***
	Year	55.47NS	4.99NS	16.19NS
	Year × genotype	7.38***	0.7***	415.03***
	Residual	2.27	3.72	554.11
	*H* ^2^ (SE)	0.849 (0.031)	0.798 (0.041)	0.899 (0.021)

*FL* days to flowering, *NI* nitrogen balance index, *PW* 100 panicle weight, *H* ^*2*^ broad sense heritability, *NS* not significant

***Significant at *p* = 0.001

*H* ^2^ was rather high for FL or PW, with *H* ^2^ > 0.8 and moderate for NI (*H* ^2^ = 0.56), in the RP. For the PP, *H* ^2^ was high for all traits (*H* ^2^ ≥ 0.8). The precision of the *H* ^2^ estimates was reasonably high, as the standard errors ranged between 0.007 and 0.052.

### Genotypic data and genetic diversity

The 43,686 SNP markers were unevenly distributed along the chromosomes. While the average marker density was 1 SNP per 8.8 kb, it ranged from one SNP every 5.1 kb on chromosome 11 to one SNP every 12.6 kb on chromosome 3 (Supplementary Table 3; Supplementary Fig. 3). The distance between a pair of adjacent SNPs ranged from 0.001 to 644 kb, with a median of 1.20 kb. The distance was < 20 kb in almost 90% of the pairs of adjacent markers and < 100 kb in 98.8%. The distance was > 100 kb in 500 pairs of adjacent SNPs, > 200 kb in 114 pairs and > 500 kb in only one pair.

Even though the markers whose MAF was below 2.5% had been discarded, the distribution of MAF was still skewed toward low frequencies. The proportion of loci with a MAF < 10% was 38.5% in the RP and 30% in the PP. The differences in allele frequency between the two populations were mainly quantitative, not qualitative: for 93% of loci, the minor allele in RP was also the minor allele in PP, and the average MAF for these loci was 24.6 in RP and 22.7 in PP. Conversely, for 7% of loci, the minor allele in RP (average frequency of 42.7%) become a major allele in PP with an average frequency of 56.4%, suggesting the accentuation of allele frequency disequilibrium observed in RP. This tendency was confirmed by the allele frequencies in loci with the lowest MAF (< 10%) in RP. In these loci, which represented 16% of the total, only one minor allele in RP became a major allele in PP, while 951 minor alleles in RP were lost in PP and the corresponding loci became monomorphic with the major allele of RP. The MAF differences between RP and PP were even more pronounced for the smallest incidence matrix of 3322 SNPs, with a MAF < 5% for 25.7% of the loci and 9.2% of monomorphic loci.

**Fig. 2:**
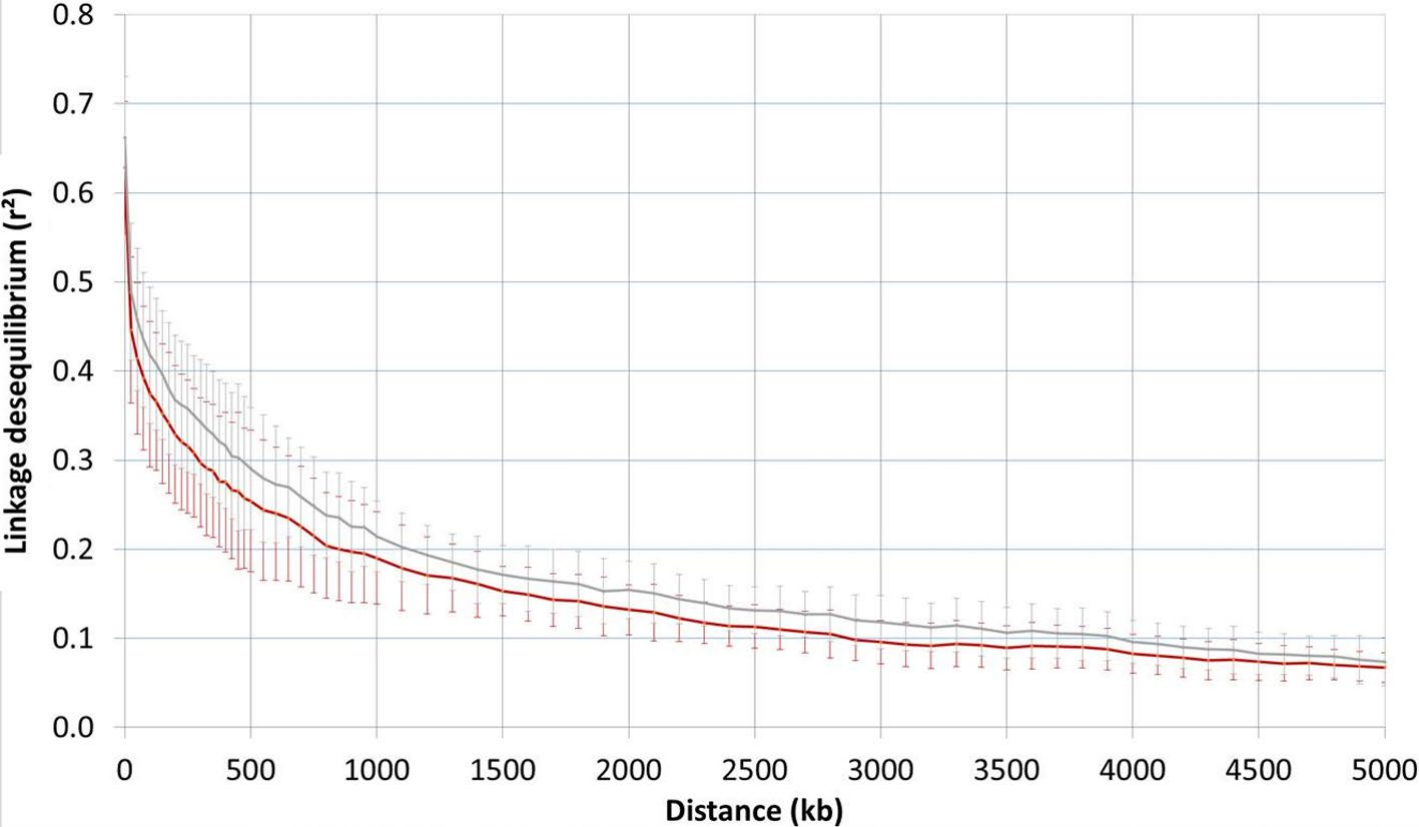
Patterns of decay in linkage disequilibrium in the reference population (red) and in the progeny population (gray). The curve represents the average *r* ^2^ among the 12 chromosomes and the bars represent the associated standard deviation (color figure online)

The decay of LD along physical distance is presented in Fig. 2 and Supplementary Table 4. For between-marker distances of 0 to 25 kb, the *r* ^2^ value reached 0.62 and 0.66 in the RP and in the PP, respectively. In the RP, the *r* ^2^ value dropped to half its initial level at around 350 kb and reached 0.2 at 800 kb and 0.1 at 2.9 Mb. As expected, the decay of LD was slower in the PP, reaching an *r* ^2^ of 0.2 at 1.1 Mb and 0.1 at 3.9 Mb. Some differences in the speed of LD decay were observed between chromosomes, with the highest speed in chromosome 11 (*r* ^2^ = 0.21) reached between 200 and 225 kb in the RP and an *r* ^2^ of 0.20 reached between 300 and 350 kb, and the lowest in chromosome 5, with an *r* ^2^ of 0.2 at 1–1.5 Mb in both populations.

**Fig. 3:**
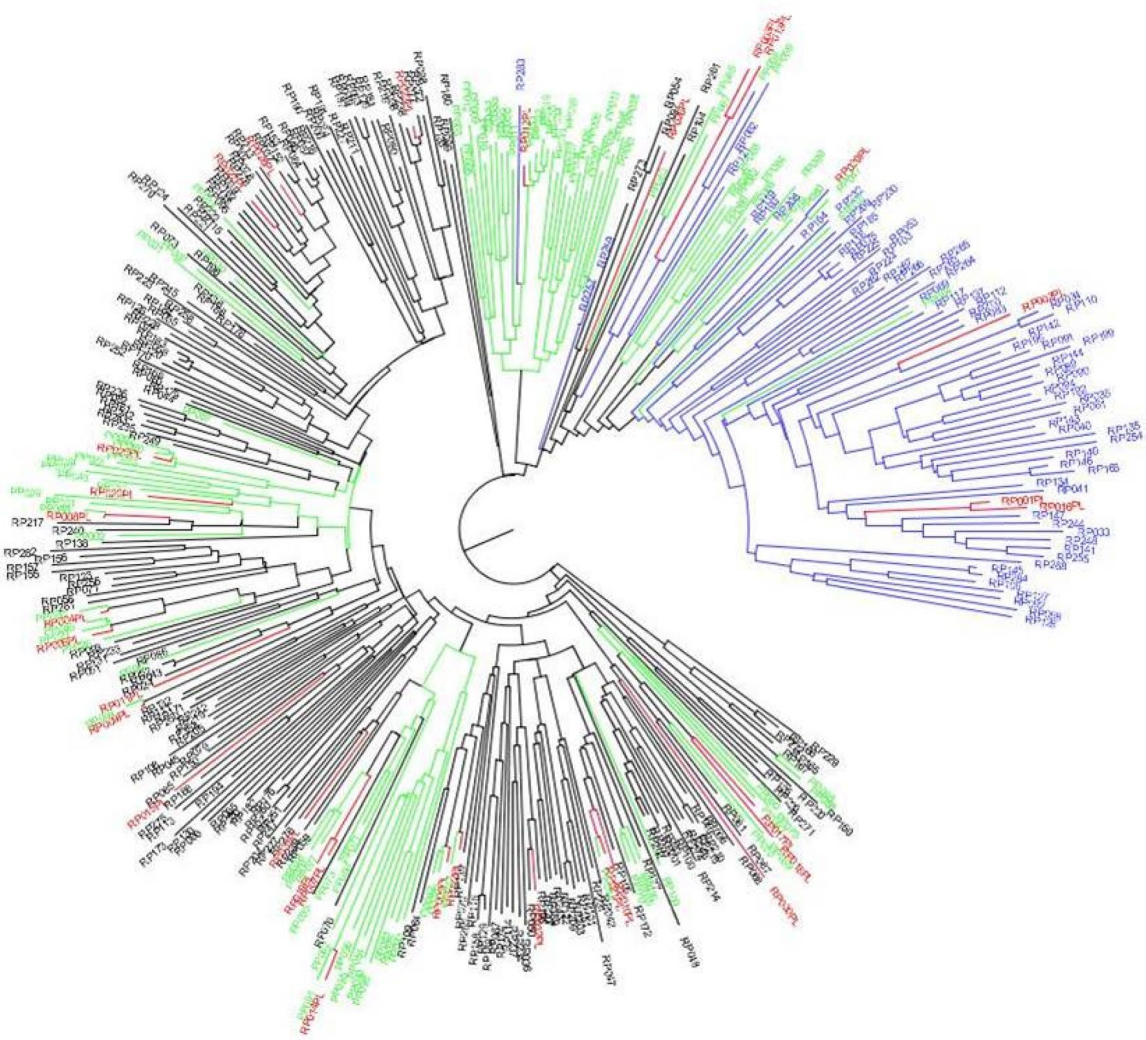
Unweighted neighbor-joining tree based on simple matching distances constructed from the genotype of 284 accessions of the reference population (RP) and 97 lines of the progeny population (PP), using 4824 SNP markers. Red: parental lines (PL); Black and blue: RP accessions belonging to tropical *japonica* and temperate *japonica*, respectively; Green: PP (color figure online)

The genetic diversity analysis of the RP led to two major clusters corresponding to the well-known temperate *japonica* (217 accessions) and tropical *japonica* (67 accessions) sub-groups (Fig. 3). The majority of the temperate *japonica* accessions are of European origin. The majority of tropical *japonica* accessions originate from the American continent. Interestingly, the average values for the three phenotypic traits investigated differed significantly in the two groups: 92 and 98 days for FL, 24.5 and 21.8 for NI and 354 and 305 g for PW in the temperate and the tropical *japonica* groups, respectively. Among the 31 accessions involved in biparental crosses for the development of the PP lines, 24 belonged to the temperate *japonica* group and seven to the tropical *japonica* group. Including the PP in the diversity analysis did not modify the clustering into two groups, but only six progeny lines clustered with the tropical *japonica* group, while out of the 97 lines, a total of 43 derived from 11 crosses involving a tropical *japonica* donor. The remaining 37 PP lines derived from crosses involving a tropical *japonica* donor clustered with the temperate *japonica* group (Fig. 3; Supplementary Table 2).

### Accuracy of genomic prediction in the diversity reference panel

**Fig. 4:**
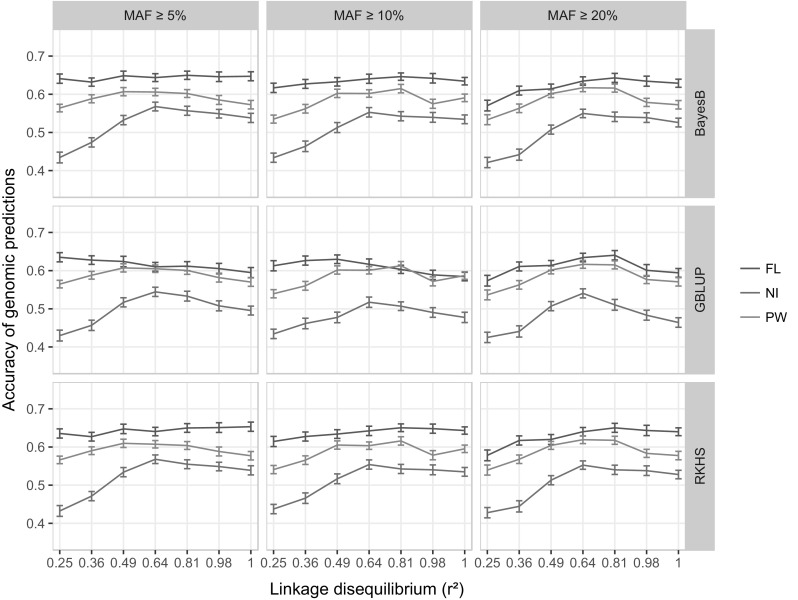
Accuracy of genomic prediction in cross validation experiments in the reference population for days to flowering (FL), nitrogen balance index (NI) and 100 panicle weight (PW), obtained with 3 statistical methods, BayesB, GBLUP and RKHS

The 189 cross validation experiments involving seven levels of LD, three levels of MAF, three prediction methods and the three phenotypic traits yielded average prediction accuracies (APA) ranging from 0.42 to 0.65 (Fig. 4; Supplementary Table 5). The overall APA for the FL trait (the average prediction accuracy over 7 LD levels × 3 MAF levels × 3 prediction methods = 63 cross validation experiments) was 0.63. The overall APA was 0.50 for NI and 0.59 for PW. Given the notable difference between traits, a model per trait was fitted to assess the effects of LD, MAF and statistical method. LD had significant effects on the APA of each trait. The MAF and method effects were significant only for FL and NI (Table 5). The LD threshold leading to the highest APA (0.60), considering the three traits, was *r* ^2^ ≤ 0.64 and *r* ^2^ ≤ 0.81. The LD threshold leading to the lowest APA (0.53 and 0.55) among the three traits was *r* ^2^ ≤ 0.25 and *r* ^2^ ≤ 0.36. NI was the trait most affected by variations in the LD threshold, with a gain in APA of 0.12 (21.6%) between LD levels (*r* ^2^ = 0.25 and *r* ^2^ = 0.64) giving the lowest, and the highest APA, respectively. The MAF threshold leading to the overall highest APA (0.58) was MAF ≥ 5%. The higher MAF thresholds tested led to the same lower APA (0.57). Finally, the performances of the BayesB and RKHS methods were the same (0.58) and that of GBLUP was 0.56.

**Table 5:** ANOVA of factors affecting the transformed accuracy (Z) of the 63 cross validation experiments per trait in the reference population

Model	Trait	*R* ^2^	CV	RMSE	Mean	Source	*df*	SS	MS	*F* value	Prob *F*
Main effects only	FL	0.5650	3.27	0.024	0.74	Model	10	0.0393	0.0039	6.75	< .0001
						Error	52	0.0302	0.0006		
						Corrected total	62	0.0695			
						LD	6	0.0127	0.0021	3.63	0.0044
						MAF	2	0.0068	0.0034	5.85	0.0051
						Method	2	0.0198	0.0099	17.02	< .0001
	NI	0.9404	2.82	0.016	0.56	Model	10	0.2014	0.0201	82.02	< .0001
						Error	52	0.0128	0.0002		
						Corrected total	62	0.2142			
						LD	6	0.1780	0.0297	120.8	< .0001
						MAF	2	0.0055	0.0028	11.22	< .0001
						Method	2	0.0179	0.0090	36.47	< .0001
	PW	0.8849	1.96	0.013	0.67	Model	10	0.0692	0.0069	39.99	< .0001
						Error	52	0.0090	0.0002		
						Corrected total	62	0.0782			
						LD	6	0.0679	0.0113	65.34	< .0001
						MAF	2	0.0009	0.0005	2.61	0.0834
						Method	2	0.0005	0.0002	1.31	0.2792
Main effects + first-order interactions	FL	0.9742	1.17	0.009	0.74	Model	38	0.0677	0.0018	23.82	< .0001
						Error	24	0.0018	0.0001		
						Corrected total	62	0.0695			
						LD	6	0.0127	0.0021	28.25	< .0001
						MAF	2	0.0068	0.0034	45.44	< .0001
						Method	2	0.0198	0.0099	132.28	< .0001
						LD × method	12	0.0126	0.0010	14.01	< .0001
						LD × MAF	12	0.0139	0.0012	15.43	< .0001
						MAF × method	4	0.0020	0.0005	6.74	0.0009
	NI	0.9938	1.34	0.007	0.56	Model	38	0.2129	0.0056	100.89	< .0001
						Error	24	0.0013	0.0001		
						Corrected total	62	0.2142			
						LD	6	0.1780	0.0297	534.32	< .0001
						MAF	2	0.0055	0.0028	49.62	< .0001
						Method	2	0.0179	0.0090	161.3	< .0001
						LD × method	12	0.0087	0.0007	13.04	< .0001
						LD × MAF	12	0.0024	0.0002	3.55	0.004
						MAF × method	4	0.0004	0.0001	1.72	0.178
	PW	0.9998	0.13	0.001	0.67	Model	38	0.0782	0.0021	2610.91	< .0001
						Error	24	0.0000	0.0000		
						Corrected total	62	0.0782			
						LD	6	0.0679	0.0113	14,349.8	< .0001
						MAF	2	0.0009	0.0005	572.6	< .0001
						Method	2	0.0005	0.0002	287.19	< .0001
						LD × method	12	0.0001	0.0000	13.55	< .0001
						LD × MAF	12	0.0088	0.0007	935.42	< .0001
						MAF × method	4	0.0000	0.0000	2.13	0.1078

*R* ^*2*^ coefficient of determination, *CV* coefficient of variation, *RMSE* root mean square error, *Mean* intercept value of the transformed accuracy (*Z*), *FL* days to flowering, *NI* nitrogen balance index, *PW* 100 panicle weight, *LD* linkage disequilibrium with 7 levels (LD ≤ 0.25, LD ≤ 0.36, LD ≤ 0.49, LD ≤ 0.64, LD ≤ 0.81, LD ≤ 0.98, LD ≤ 1, *MAF* minor allele frequency with 3 levels (MAF ≥ 5%, MAF ≥ 10%, MAF ≥ 20%), *Method* BayesB, GBLUP, RKHS

All first rank interactions between the three factors affecting APA were significant except MAF × Method for NI and PW traits (Table 5). Among the three prediction methods, GBLUP was the most affected by the level of LD (Supplementary Fig. 4A). Indeed, for the LD threshold higher than 0.64, RKHS and BayesB performed significantly better than GBLUP with an increase in accuracy of up to 0.04. In particular, this was the case of the FL and NI traits. The MAF × LD interaction led to diverging accuracies between MAF thresholds under the most stringent LD thresholds of *r* ^2^ ≤ 0.49 (Supplementary Fig. 4B).

Given these results, we decided to consider only one MAF threshold (≥ 5%) in the following steps of the study (progeny prediction) and to focus on the analysis of the effect of LD and prediction method.

### Accuracy of genomic prediction across generations

**Fig. 5:**
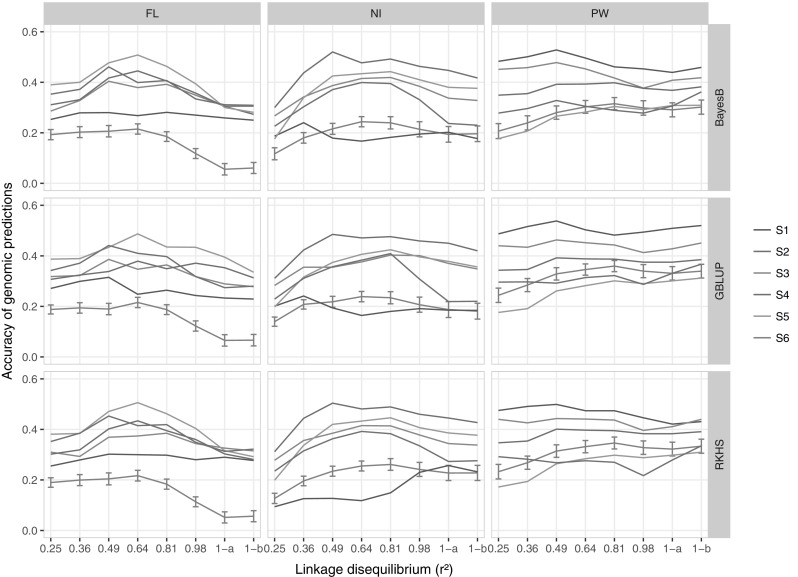
Accuracy of genomic prediction of progeny phenotype for days to flowering (FL), nitrogen balance index (NI) and 100 panicle weight (PW), obtained with three statistical methods, BayesB, GBLUP and RKHS. The six scenarios are described in Table 3. For scenario S6 that includes random sampling, the average and the 95% confidence interval are shown. 1-a and 1-b, represent incidence matrices with no selection on *r* ^2^, but filtered with MAF > 5% and MAF > 2.5%, respectively

The 360 non-replicated experiments of genomic prediction of the progenies’ phenotype, involving the first five scenarios (S1 – S5) of the relationship between the training set and the progeny set, seven LD thresholds, and three prediction methods led to progeny prediction accuracies (PPA) ranging from 0.23 to 0.51 (mean PPA 0.35) for the FL trait, 0.09 to 0.52 (mean PPA 0.33) for NI and 0.17 to 0.54 (mean PPA 0.38) for PW. The 72 replicated prediction experiments in scenario S6 led to an average PPA ranging from 0.05 to 0.22 (mean PPA 0.15) for the FL trait, 0.12 to 0.26 (mean PPA 0.21) for NI and 0.21 to 0.36 (mean PPA 0.30) for PW. The following comparisons of factors affecting PPA, especially the scenario factor, are based on PPA data for the non-replicated prediction experiments and on the average PPA for the replicated experiments of S6 (Fig. 5; Supplementary Table 6).

Variation in the scenario and in the LD factors significantly affected PPA for all traits. The effect of the prediction method was not significant (Table 6). The effects of interactions between scenario and LD and scenario and method were significant for the three traits. The interaction between LD and method was significant only for the FL trait. These interactions limit the interpretation of the individual main effects. Nevertheless, it is noteworthy that of the three factors, the scenario effect showed the greatest variances for the three phenotypic traits under the two ANOVA models, and, as expected, the trend of variations in PPA was related to both the size and the degree of relatedness of the training set with the progeny set. Consequently, for each trait, the mean PAA obtained with the medium size training sets S3 (0.34, 0.36 and 0.43, for FL, NI and PW, respectively) was not much below the one obtained with the largest training set S4 (0.35, 0.44 and 0.40, for FL, NI and PW, respectively). The presence of the parental line in the training set improved the PPA for NI and PW, as illustrated by the lower mean PPA of NI and PW in S2 (0.31), compared to the mean PPA of NI and PW in S3 (0.40), and the mean PPA of NI and PW in S5 (0.32), compared to S4 (0.42). This was not the case for FL (mean PPA of 0.37 in S2 and 0.34 in S3; mean PPA of 0.35 in S4 and 0.41 in S5), probably because the three prediction models we used could not capture the transgressive distribution of this trait observed in the progeny of the same crosses. Likewise, when the size of the training set was too small, like in S1 (training set = 31 parental lines; mean PPA = 0.31), good relatedness was not sufficient to obtain a similar PPA to that obtained with a larger training set.

**Table 6:** ANOVA of factors influencing the transformed accuracy (*Z*) of 126 progeny prediction experiments for three phenotypic traits

Model	Trait	*R* ^2^	CV	RMSE	Mean	Source	*df*	SS	MS	*F* value	Prob *F*
Main effects only	FL	0.855	14.64	0.036	0.25	Model	13	0.862	0.066	50.77	< .0001
						Error	112	0.146	0.001		
						Corrected total	125	1.008			
						LD	6	0.188	0.031	24	< .0001
						Method	2	0.003	0.002	1.22	0.2999
						Scenario	5	0.671	0.134	102.72	< .0001
	NI	0.848	12.48	0.045	0.36	Model	13	1.277	0.098	48.19	< .0001
						Error	112	0.228	0.002		
						Corrected total	125	1.505			
						LD	6	0.237	0.040	19.41	< .0001
						Method	2	0.000	0.000	0.03	0.968
						Scenario	5	1.039	0.208	102	< .0001
	PW	0.876	12.17	0.047	0.39	Model	13	1.791	0.138	61.11	< .0001
						Error	112	0.252	0.002		
						Corrected total	125	2.043			
						LD	6	0.035	0.006	2.6	0.0213
						Method	2	0.003	0.001	0.6	0.5497
						Scenario	5	1.753	0.351	155.53	< .0001
Main effects + first-order interactions	FL	0.978	7.79	0.019	0.25	Model	65	0.986	0.015	40.99	< .0001
						Error	60	0.022	0.000		
						Corrected total	125	1.008			
						LD	6	0.188	0.031	84.68	< .0001
						Method	2	0.003	0.002	4.3	0.018
						Scenario	5	0.671	0.134	362.49	< .0001
						LD × method	12	0.014	0.001	3.06	0.002
						LD × scenario	30	0.102	0.003	9.15	< .0001
						Method × scenario	10	0.009	0.001	2.4	0.0181
	NI	0.980	6.18	0.022	0.36	Model	65	1.475	0.023	45.45	< .0001
						Error	60	0.030	0.000		
						Corrected total	125	1.505			
						LD	6	0.237	0.040	79.21	< .0001
						Method	2	0.000	0.000	0.13	0.8761
						Scenario	5	1.039	0.208	416.31	< .0001
						LD × method	12	0.006	0.001	1.01	0.4507
						LD × scenario	30	0.173	0.006	11.57	< .0001
						Method × scenario	10	0.019	0.002	3.78	0.0006
	PW	0.972	7.93	0.031	0.39	Model	65	1.986	0.031	31.97	< .0001
						Error	60	0.057	0.001		
						Corrected total	125	2.043			
						LD	6	0.035	0.006	6.14	< .0001
						Method	2	0.003	0.001	1.42	0.2499
						Scenario	5	1.753	0.351	366.83	< .0001
						LD × method	12	0.012	0.001	1.04	0.4256
						LD × scenario	30	0.120	0.004	4.18	< .0001
						Method × scenario	10	0.063	0.006	6.62	< .0001

*R* ^*2*^ coefficient of determination, *CV* coefficient of variation, *RMSE* root mean square error, *Mean* intercept value of the transformed accuracy (*Z*), *FL* days to flowering, *NI* nitrogen balance index, *PW* 100 panicle weight, *LD* linkage disequilibrium with 7 levels (LD ≤ 0.25, LD ≤ 0.36, LD ≤ 0.49, LD ≤ 0.64, LD ≤ 0.81, LD ≤ 0.98, LD ≤ 1), *MAF* minor allele frequency with 3 levels (MAF ≥ 5%, MAF ≥ 10%, MAF ≥ 20%, *Method* BayesB, GBLUP, RKHS

To further explore the effect of relatedness between the training and the candidate set, we used the CDmean method (Rincent et al. 2012) to select the accessions to be included in the training set. Three training sets (*n* = 31, *n* = 58 and *n* = 89, equivalent in size to scenarios S1, S2 and S3, respectively) were selected using the CDmean method, and used to predict progeny. The results (Supplementary Fig. 5) showed almost no gain in accuracy for FL and PW with the CDmean method, and an almost systematic gain in accuracy of about 0.1 for NI.

The magnitude of variation in PPA in relation with LD was much narrower. The highest mean PPA (0.36) was achieved with LD thresholds of *r* ^2^ ≤ 0.49 to *r* ^2^ ≤ 0.81, when interactions with other factors were left aside. The PPA decreased smoothly with both lower and higher LD thresholds, and reached 0.28 for *r* ^2^ ≤ 0.25, and 0.31 for *r* ^2^ ≤ 1. The inclusion of additional markers under *r* ^2^ ≤ 1, by lowering the MAF threshold to 2.5%, neither deteriorated nor improved the PPA (Supplementary Table 6).

As expected, the accuracy of progeny prediction within two individual crosses showed much larger variation (− 0.310 to 0.731) depending on the trait, the scenario and the size of the incidence matrix (Supplementary Figs. 6 and 7). Given the small number of progenies for each of the two crosses used for this analysis (20 and 9), drawing any conclusion regarding the effect of one individual factor (scenario, trait, LD) would be risky. However, the fact that accuracy above 0.7 was obtained in some conditions strongly suggests the feasibility of intra-cross progeny prediction using a diversity panel.

## Discussion

The main objectives of this work were to assess the performance of genomic prediction among the progeny of biparental crosses, using a reference panel to train the model in rice, and to investigate the effect of the size of the reference panel and of the degree of relatedness with the progeny population on the accuracy of predictions, as well as the effect of LD and the training model. To set a base line for prediction accuracy and to reduce the number of possible options to be tested regarding LD and other characteristics of the incidence matrix, we started our study by evaluating the accuracy of genomic prediction within the reference population using a cross validation approach.

### Accuracy of genomic prediction in the reference population

The average genomic prediction accuracies within the reference population ranged from 0.51 for NI to 0.63 for FL, in line with their degree of broad sense heritability. The highest accuracies were 0.65 for FL, 0.57 for NI, and 0.62 for PW. Beyond the high heritability, the rather narrow genetic diversity of our RP assembling temperate and tropical *japonica* adapted to the irrigated lowland ecosystem of Europe, has probably contributed to the relatively high accuracy of genomic prediction for complex traits such as NI and PW.

The accuracy of genomic prediction was affected in a complex way by interactions between LD, MAF and phenotypic traits, but did not question the well-established rule of balance between the number and the distribution of markers along the chromosome, and the LD within the population (Jannink et al. 2010). However, the GBS genotyping method resulted in heterogeneous marker distribution, with distances between adjacent marker varying from one base to more than one Mb. The pruning of SNP markers based on LD information enabled us to improve accuracy with non-redundant SNP matrices. Our interpretation of the increase in prediction accuracy when marker redundancy was reduced is that: (1) the higher the number of redundant markers, the smaller the contribution of individual markers in the prediction model, including those tightly linked to the QTL or the most determining one for the calculation of genomic distance between individuals; (2) prediction models based on a high number of redundant markers are less accurate, as they capture numerous false genotype–phenotype relationships or build less discriminant genomic distances.

The trend towards an increase in prediction accuracy with a reduction in marker redundancy was best captured with GBLUP, raising the question of its origin, model formulation per se or method of implementation. Using the BGLR package (Pérez and de los Campos 2014) to fit GBLUP in a Bayesian framework with MCMC sampling (GBLUP_B), we explored the hypothesis of method of implementation. Our results revealed (Supplementary Fig. 8) a difference between the two methods with a plateau of accuracy for GBLUP_B for matrices with medium to high marker density, suggesting that the implementation of the GBLUP method was responsible of the decrease of accuracy rather than the method itself. This difference is probably related to a better convergence of the MCMC algorithm compared to the EM algorithm.

In the present study, we used a simple procedure based on pairwise LD to eliminate the most redundant markers. Other procedures have been developed: selection of tag SNPs based on LD, diversity or hot spots of recombination (Carlson et al. 2004; Zhang et al. 2004; Halperin et al. 2005), measuring the contribution of each marker with a statistic called ‘degree of tagging’, that includes both pairwise LD and base-pair distance (Speed et al. 2012; Ramstein et al. 2016). The practical lesson that can be drawn from our procedure is that the accuracy of prediction can be significantly improved by not including markers that constitute the largest high redundancy clusters.

Compared to LD, the MAF and the prediction method had much more limited effects on prediction accuracy, although the effects were significant. The rather small MAF effect suggests that low MAF mainly results from random genetic drift (Edriss et al. 2012) and/or that markers closely linked with genes which affect our target traits, have not yet reached a high level of fixation within our population. Regarding the method effect, the lower performances of GBLUP for predicting FL and NI suggests the existence of QTLs with a rather large effect that could be better captured using methods based on marker effect than using information on genomic relationships.

### Accuracy of genomic prediction of progeny performances

Our genomic prediction experiments on the line value of F_5_-F_7_ progenies of biparental crosses, each involving two accessions belonging to the reference population, mimicked a rice breeding scheme in which the breeding cycle is shortened by rapid generation advancement (RGA) of the early generations, and where the phenotypic evaluation starts with the advanced F_5_ or F_6_ generation. RGA consists in the fixation of F_2_ progenies through 2–3 generations of single seed descent per year in the greenhouse, until F_5_ or F_6_. However, our experiments diverged from this scheme by the very pronounced imbalance in the number of progeny per cross, which varied from 1 to 20.

The accuracy of our progeny predictions among the 97 advanced lines of PP derived from 36 biparental crosses, involving 31 accessions of RP, varied greatly for each trait, depending on the composition of the training set and the LD. However, for each trait, the highest degree of accuracy achieved was only slightly below the highest accuracy achieved in the cross validation experiments in the RP: 0.51 versus 0.65 for FL, 0.52 versus 0.57 for NI and 0.54 versus 0.62 for PW. Similar results have been obtained in sugar beet (Hofheinz et al. 2012), in barley (Sallam et al. 2015), in wheat (Michel et al. 2016) and in strawberry (Gezan et al. 2017). What is more, in our case, rather high accuracies (up to 0.7) were obtained in progeny prediction among the full-sib lines of individual crosses. However, the number of progeny per cross and the number crosses analyzed were too small to draw general conclusions.

Population parameters that affect the accuracy of progeny prediction include differences in LD and allele frequency between RP and PP, as well as the parental contributions to PP and the genetic distance, or number of generations, between the two populations (Daetwyler et al. 2010; Lorenz et al. 2012). Recombination in breeding populations reduces LD between markers and QTLs over time, while selection increases LD (Pfaffelhuber et al. 2008). In our case, only one cycle of recombination separated RP and PP and the two populations did not differ much in either long distance LD or short distance LD, i.e., LD between markers and QTLs (Fig. 2). This is not particularly surprising given the composition of PP, involving a large number of biparental crosses. Moreover, the average LD (*r* ^2^ = 0.2 at 850 and 1100 kb distance in RP and PP, respectively) was much higher than that reported in the literature for the *japonica* group (Courtois et al. 2013), suggesting narrower genetic diversity of the RP compared to the whole *japonica* group. Regarding the genetic distance between RP and PP, and the contributions of individual parental lines to the final composition of PP, marked unbalance was observed, to the advantage of the temperate *japonica* subgroup. Indeed, while the tropical *japonica* subgroup represented 24% of the accessions of RP, only 8% of PP lines clustered with the tropical *japonica* subgroup. Likewise, at the level of individual crosses, voluntary or involuntary selection of progenies skewed their distribution toward the temperate *japonica* genetic background. Indeed, while 38% of the 36 biparental crosses involved a tropical *japonica* accession of RP and produced more than 50% of the progeny lines of PP, only 15% of these progeny clustered with the tropical *japonica* subgroup. These unbalances raise the question of how to choose the individuals that make up the training set to maximize the accuracy of progeny predictions.

### Selection of the training set to optimize accuracy of progeny prediction

Several studies have shown that the accuracy of genomic predictions is highly influenced by the degree of relatedness between TP and CP (Pszczola et al. 2012; Rincent et al. 2012; Hayes et al. 2009b). As discussed above, in our study, there was marked variation in the degree of relatedness between the individuals of the two populations. This large variation raised the question of the choice of the RP individuals to be included in the training set to maximize the accuracy of progeny predictions. The results of the six compositions of the training set scenarios we tested confirmed the complementary effects of relatedness between the training set and the PP, and the size of the training set. The lower mean PPA observed under scenario S1, compared to scenarios S3 and S4 shows that, in addition to relatedness between the training set and PP, the size of the training set also matters, and even distant accessions can positively contribute to prediction accuracy. The results of scenario S2 demonstrate that high APA can be achieved without the presence of the parental lines in the training set provided it is composed of individuals closely related to the parental lines. The highest APA observed under scenario S4 suggests there is still room for optimization of the size and the composition of the training set. For instance, by weighting the contribution of each parental line to the composition of the pools of the most closely and most distantly related individuals in the RP, based on their actual contribution (ratio of the number of progeny to the total number of individuals in the PP) to the composition of the PP. The almost equal APA observed in S3 and S4 suggests that beyond a certain size threshold of the training set composed of accessions closely related to the PP, the inclusion of less closely related individuals does not improve prediction accuracy. These findings are in agreement with those of Pszczola et al. (2012), who showed that the relatedness between the reference individuals and between the candidates and the reference individuals has a strong effect on accuracy. Given the above-mentioned effects of selection on PP, one could expect better prediction accuracy with optimization methods that directly use information on relatedness between the individuals in the training set and the individuals in the PP, such as CDmean (Rincent et al. 2012). Comparison of accuracy obtained under scenarios S1, S2 and S3, with the accuracy obtained with the training set selected using the CDmean method only partially confirmed this expectation. This is probably due to the fact that our scenario for optimization of the training set was also based on relatedness between the training set and the parental lines of the PP.

All our training set optimization experiments targeted genomic prediction among the progenies of 36 biparental crosses. In general, pedigree breeding programs target the progeny of individual biparental crosses. Most of the individual biparental populations in our data set were too small for such experiments. However, prediction accuracies of above 0.5 were observed for NI and WP under some LD thresholds and in some scenarios, even though the training sets were not specifically optimized for these populations. This encouraging result merits confirmation using a more appropriate data set.

When predicting GEBVs on progeny, the optimal size of the training set depends on the degree of relatedness (number of generations between the training set and the progeny set), the effective size of population Ne, the length of the genetic map, and the architecture of the target trait (Jannink et al. 2010). Generally speaking, an increase in the size of the TP improves prediction accuracy, but in addition to size, the genetic structure of the TP and the relationship between this structure and the distribution of the target trait, also matter. For instance, Technow et al. (2013) observed a 10% increase in prediction accuracy, when they combined data from two heterotic groups of corn (flint and dent) to predict resistance to leaf blight in one of the groups. Conversely, Lorenz et al. (2012) observed no significant improvement in the prediction of resistance to fusarium head blight and its associated resistance to mycotoxins, when they increased the size of the TP by combining different barley breeding populations. In the present study, the highest average accuracies were achieved with the largest training set for PW and NI traits that have complex genetic architecture. Prediction accuracy was less responsive to the size of the training set for the FL trait, of oligo-genic determinism (Hori et al. 2016).

### Practical implications for rice breeding programs

Pedigree breeding within the progenies of biparental crosses extracted from a working collection or reference population is the most common scheme for the improvement of complex traits in rice, as in many other autogamous crops (Bernardo 2014). We found that, using phenotypic and genotypic data from the RP to train the prediction model made it possible to predict performances among the first generation of advanced (F_5_–F_7_) progeny of a large set of biparental crosses. Accuracies of over 0.5 were obtained, even for complex traits such as NI and PW, when the parameters that affect the accuracy were optimized. Thus, breeders can use this prediction approach in the framework of a pedigree breeding scheme associated with RGA of early generations (in off-season nurseries or controlled environments), a practice aimed at reducing the length of the breeding cycle and hence accelerating genetic gain per unit of time (O’Connor et al. 2013). However, specific optimization of the training set might be needed to obtain the best possible prediction accuracy for the progeny of each cross. The scheme can also be applied in breeding schemes that use the haplo-diploidization method for the rapid generation of homozygous lines from biparental crosses, at least in the *japonica* genetic group for which a high-throughput haplo-diploidization method is available (Alemanno and Guiderdoni 1994). As the advanced line selected in this way will then go through 2–3 cycles of phenotypic evaluation, the data collected will provide an opportunity to further refine the training model (Heffner et al. 2010).

We also found that (1) an average marker density above one per 22 kb (8324 SNPs) did not improve the accuracy of prediction in either cross validation within the RP or in progeny prediction and (2) relatively high accuracy could be achieved using only a rather small share of the RP, most related to PP, as the training set. Given the very uneven distribution of marker density along the chromosomes in our RP and PP, one would expect similar levels of prediction accuracy with a much smaller number of markers chosen based on LD distribution along the chromosomes, as already predicted in simulation studies (Habier et al. 2009; Lillehammer and Meuwissen 2013; Grattapaglia 2014). These findings attest to the feasibility of using the genomic selection approach in breeding programs with rather limited resources. The most efficient and affordable option would be rather dense genotyping of the RP accessions and much looser (a few hundred), but evenly distributed, genotyping of PP that can be densified through imputation, a method widely practiced in animal breeding (Marchini and Howie 2010).

#### Author contributions

NA and GV conceived the study. CC extracted the DNA and prepared the library for GBS. GO, JR, LR, CB, CB, AV, FD, MBH implemented the field trials. MBH, TVC, JB, LJ, NA analyzed the data. MBH and NA wrote the manuscript. TVC and JB revised the manuscript.

## Electronic supplementary material

Below is the link to the electronic supplementary material.

Supplementary material 1 (PDF 386 kb)

Supplementary material 2 (PDF 221 kb)

Supplementary material 3 (PDF 620 kb)

Supplementary material 4 (PDF 614 kb)

Supplementary material 5 (PDF 612 kb)

Supplementary material 6 (PDF 223 kb)

Supplementary material 7 (JPEG 124 kb)

Supplementary material 8 (JPEG 692 kb)

Supplementary material 9 (JPEG 85 kb)

Supplementary material 10 (JPEG 98 kb)

Supplementary material 11 (JPEG 156 kb)

Supplementary material 12 (JPEG 138 kb)

Supplementary material 13 (JPEG 141 kb)

Supplementary material 14 (JPEG 116 kb)
