# Supplementary material for: Rice diversity panel provides accurate genomic predictions for complex traits in the progenies of biparental crosses involving members of the panel
Source: Theor Appl Genet. 2017 Nov 14;131(2):417–35. doi: 10.1007/s00122-017-3011-4 (PMC5787227; doi:10.1007/s00122-017-3011-4)
Supplement: Supplementary file 2 — Supplementary material 2 (PDF 221 kb) [file 122_2017_3011_MOESM2_ESM.pdf]

Supplementary Table 2: List of the 97 F5-F7 lines of the progeny population and their phenotype for days to flowering (FL), nitrogen balance index (NI) and 100 panicles weight (PW).

Genotype	Crosses	FL (days)	NI	PW (g)
PF043	Aiace / Perla	97.83	15.42	184.71
PF048	Apollo / Volano	96.50	15.64	272.91
PF031	Apollo/Selenio//Apollo	92.33	17.14	216.46
PF081	Apollo/Selenio//Apollo	94.00	16.76	255.47
PF057	Asia / 2*Selenio	94.83	20.34	316.05
PF058	Asia / 2*Selenio	94.83	19.00	195.4
PF059	Asia / 2*Selenio	107.00	20.86	210.07
PF085	Asia / Centauro	97.00	17.23	182.2
PF087	Asia / Centauro	96.33	17.11	243.27
PF030	Augusto / Gigante Vercelli	80.17	22.66	281.54
PF069	Augusto / Handao 297	94.67	20.38	173.15
PF070	Augusto / Handao 297	95.33	19.95	227.75
PF042	Baldo / Handao 297	91.50	19.29	398.7
PF052	Baldo / Handao 297	85.50	22.22	303.79
PF053	Baldo / Handao 297	90.67	18.91	285.46
PF055	Baldo / Handao 297	90.17	17.72	284.33
PF019	Baldo / Opale	90.67	17.58	292.24
PF020	Baldo / Opale	91.67	18.93	380.09
PF021	Baldo / Opale	96.17	17.77	328.84
PF022	Baldo / Opale	90.33	18.78	274.58
PF023	Baldo / Opale	83.83	17.45	278.17
PF024	Baldo / Opale	90.83	18.55	286.5
PF015	Carmen / Creso	95.00	18.17	323.01
PF016	Carmen / Creso	93.50	17.71	301.36
PF017	Carmen / Creso	95.50	16.32	325.19
PF026	Carmen / Loto	89.26	17.28	276.74
PF027	Carmen / Loto	88.83	19.63	182.44
PF068	Centauro / Dimitra	89.00	20.94	259.01
PF051	Centauro / Koral	83.33	21.77	289.97
PF071	Centauro / Koral	100.17	23.66	286.79
PF072	Centauro / Koral	86.33	20.91	254.45
PF073	Centauro / Koral	99.83	23.96	280.09
PF047	Creso / Apollo	91.50	18.25	259.26
PF001	Delfino / 2*Centauro	88.67	18.84	248.21
PF002	Delfino / 2*Centauro	92.00	19.18	256.57
PF067	Delfino / Centauro	95.00	21.56	342.31
PF100	Delfino / Selenio	84.00	19.12	196.06
PF084	Eurosis / Gladio	88.67	15.75	233.59
PF004	Eurosis / Handao 11	88.00	18.17	303.74
PF005	Eurosis / Handao 11	86.00	12.21	228.03
PF006	Eurosis / Handao 11	90.93	18.91	320.01
PF007	Eurosis / Handao 11	97.67	19.17	293.16
PF008	Eurosis / Handao 11	102.50	18.19	308.41

PF009	Eurosis / Handao 11	96.83	15.53	245.35
PF010	Eurosis / Handao 11	102.50	14.59	240.41
PF011	Eurosis / Handao 11	95.00	17.18	299.83
PF012	Eurosis / Handao 11	92.83	17.37	363.48
PF013	Eurosis / Handao 11	101.50	17.72	298.86
PF028	Eurosis / Handao 11	83.67	18.31	240.69
PF032	Eurosis / Handao 11	108.83	19.14	256.69
PF033	Eurosis / Handao 11	90.50	25.13	247.38
PF034	Eurosis / Handao 11	96.33	16.38	240.44
PF035	Eurosis / Handao 11	88.83	18.90	287.98
PF036	Eurosis / Handao 11	92.83	20.43	248.68
PF037	Eurosis / Handao 11	91.00	20.33	241.5
PF038	Eurosis / Handao 11	91.33	14.71	266.5
PF039	Eurosis / Handao 11	98.00	15.85	304.32
PF046	Eurosis / Handao 11	87.67	15.57	283.06
PF082	Fragrance / Karnak	107.50	21.35	219.11
PF091	Giano / Loto	89.50	18.37	155.66
PF025	Giano / Vialone Nano	90.83	17.84	391.12
PF054	Giano / Vialone Nano	86.33	15.41	222.66
PF092	Giano / Vialone Nano	89.83	18.95	395.66
PF093	Giano / Vialone Nano	90.50	19.66	397.67
PF094	Giano / Vialone Nano	94.17	18.59	268.45
PF095	Giano / Vialone Nano	93.33	20.08	210.72
PF096	Giano / Vialone Nano	90.17	16.89	243.39
PF097	Giano / Vialone Nano	91.33	19.50	267.96
PF098	Giano / Vialone Nano	94.17	20.64	219.17
PF060	Gladio / Eurosis /Gladio	90.00	16.62	384.51
PF061	Gladio / Opale	96.50	16.63	276.44
PF062	Gladio / Opale	89.33	17.80	417.21
PF063	Gladio / Opale	88.83	14.40	319.45
PF064	Gladio / Opale	98.17	14.49	219.05
PF065	Gladio / Opale	92.67	17.17	341.53
PF066	Gladio / Opale	97.17	18.15	292.72
PF049	Handao 297 / Luxor	89.33	18.73	322.09
PF050	Handao 297 / Luxor	98.17	20.34	275.73
PF040	Karnak / 2*Giano	94.67	18.17	290.98
PF041	Karnak / 2*Giano	96.00	20.00	304.77
PF076	Karnak / Delfino	95.67	18.12	354.78
PF077	Karnak / Delfino	95.33	19.48	349.45
PF078	Karnak / Delfino	95.67	18.57	337.78
PF075	Karnak / Giano	96.33	17.23	292.19
PF088	Karnak / Giano	96.50	17.36	207.7
PF056	Loto / Karnak	93.33	18.88	288.34
PF044	Maratelli / Carmen	84.17	20.52	269.72
PF045	Maratelli / Carmen	85.67	20.44	273.15
PF083	Pecos / Delfino	89.50	17.10	322.59
PF014	Pecos / Gladio	92.00	19.43	221.21
PF099	Pecos / Gladio	101.83	18.14	234.23

PF089	SIS R215 / Carnaroli	91.17	18.32	239.18
PF090	SIS R215 / Carnaroli	87.17	19.45	235.35
PF079	SIS R215 / Loto	90.67	19.08	337.12
PF080	SIS R215 / Loto	91.42	16.22	305.25
PF029	Tejo / Aiace	90.50	19.30	219.68
PF074	Tejo / Centauro	93.83	23.48	330.89
