# Supplementary material for: Rice diversity panel provides accurate genomic predictions for complex traits in the progenies of biparental crosses involving members of the panel
Source: Theor Appl Genet. 2017 Nov 14;131(2):417–35. doi: 10.1007/s00122-017-3011-4 (PMC5787227; doi:10.1007/s00122-017-3011-4)
Supplement: Supplementary file 3 — Supplementary material 3 (PDF 620 kb) [file 122_2017_3011_MOESM3_ESM.pdf]

Supplementary Table 1: List of the 284 accessions of the reference population and their main characteristics.

ID	Name	Origin	Class	Year of registration	Group	FL (days)	NI	PW (g)	Parental line of PP	Most related to PP parent
TP001	AIACE	Italy	long A	2003	J-Trop	89.17	24.83	224.93	1	
TP002	APOLLO	Italy	long B	2002	J-Trop	91.50	25.51	290.96	1	
TP003	ASIA	Italy	long B	2002	J-Trop	100.83	25.05	256.55	1	
TP004	AUGUSTO	Italy	long A	2002	J-Temp	86.67	23.05	325.11	1	
TP005	BALDO	Italy	long A	1977	J-Temp	93.33	23.38	493.42	1	
TP006	CARMEN	Italy	long A	2005	J-Temp	84.33	25.10	320.91	1	
TP007	CARNAROLI	Italy	long A	1983	J-Temp	100.50	25.86	453.83	1	
TP008	CENTAURO	Italy	round		J-Temp	87.00	26.76	253.37	1	
TP009	CRESO	Italy	long A		J-Temp	92.33	25.55	345.35	1	
TP010	DELFINO	Italy	long A	2001	J-Temp	89.00	26.28	328.77	1	
TP011	DIMITRA	Greece	long A		J-Temp	95.17	24.38	366.35	1	
TP012	EUROSIS	Italy	long B		J-Trop	91.33	24.34	345.08	1	
TP013	FRAGRANCE	Italy	long B		J-Trop	93.33	28.91	250.71	1	
TP014	GIANO	Italy	long B	2003	J-Temp	91.67	25.23	222.93	1	
TP015	GIGANTE VERCELLI	Italy	long A	1967	J-Temp	94.67	25.23	498.52	1	
TP016	GLADIO	Italy	long B	1998	J-Trop	88.17	23.46	256.84	1	
TP017	HANDAO 11	China	round		J-Temp	81.50	25.14	167.02	1	
TP018	HANDAO 297	China	round		J-Temp	94.50	27.04	382.14	1	
TP019	KARNAK	Italy	long A	2002	J-Temp	99.83	24.85	420.44	1	
TP020	KORAL	Italy	long A	1981	J-Temp	91.17	26.16	427.84	1	
TP021	LOTO	Italy	long A	1988	J-Temp	85.83	24.45	272.71	1	
TP022	LUXOR	Italy	long A	2008	J-Temp	97.33	25.66	427.48	1	
TP023	MARATELLI	Italy	medium	1919	J-Temp	91.33	24.80	416.15	1	
TP024	NEMBO	Italy	long A	1999	J-Temp	87.67	28.20	370.04		1
TP025	OPALE	Italy	long A	2008	J-Temp	89.00	26.50	449.75	1	
TP026	PECOS	USA	medium		J-Temp	99.50	22.29	411.32	1	
TP027	PERLA	Italy	round	1998	J-Temp	91.83	23.75	258.56	1	
TP028	SELENIO	Italy	round	1987	J-Temp	90.17	25.35	336.00	1	
TP029	SIS R215	Italy	long A	2002	J-Trop	90.67	24.02	325.39	1	
TP030	TEJO	Italy	long A	1999	J-Temp	92.67	24.16	311.97	1	
TP031	VIALONE NANO	Italy	medium	1967	J-Temp	91.83	25.11	395.09	1	
TP032	VOLANO	Italy	long A	1972	J-Temp	95.17	25.28	349.97	1	
TP033	A201	USA	long B		J-Trop	100.33	19.86	277.11		1
TP034	A301	USA	long B	1987	J-Trop	106.67	23.40	229.51		1
TP035	ADAIR	USA	long B	1993	J-Trop	101.67	26.66	327.86		
TP036	ADELAIDE CHIAPPELLI	Italy	long A		J-temp	85.54	24.51	265.95		
TP037	AGATA	Italy	round	2012	J-Temp	93.50	26.90	300.51		1
TP038	AGOSTANO	Italy	long A	1933	J-Temp	85.50	23.03	363.20		1
TP039	AKITAKOMACHI	Japon	round		J-Temp	92.83	21.30	234.48		1
TP040	ALAN	USA	long B		J-Trop	97.00	17.30	357.33		
TP041	ALEXANDROS	Greece	long B		J-Trop	100.83	19.85	256.60		
TP042	ALICE	Italy	long A	1996	J-Temp	89.83	24.28	388.02		

TP043	ALLORIO	Italy	long A	1915	J-Temp	88.33	22.88	373.31	1
TP044	ALPE	Italy	long A	1993	J-Temp	81.33	24.42	290.28	
TP045	ALPHA	Italy	long A	1979	J-Temp	88.17	22.59	301.94	
TP046	AMERICANO 1600	Italy	round	1904	J-Temp	92.67	21.52	341.21	1
TP047	ANSEATICO	Italy	long A	1972	J-Temp	96.83	25.59	351.35	
TP048	ANTARES				J-Temp	95.50	30.78	280.59	
TP049	ANTONI	Bulgary	long A		J-Temp	76.33	18.94	220.37	
TP050	ARBORIO	Italy	long A	1967	J-Temp	95.17	23.84	451.13	1
TP051	ARGO	Italy	medium	1978	J-Temp	94.17	20.67	427.20	
TP052	ARIETE	Italy	long A	1985	J-Temp	92.00	25.63	358.59	1
TP053	ARSENAL	Italy	long B		J-Trop	92.50	26.15	252.37	
TP054	ARTEMIDE	Italy	long B		J-Temp	95.00	20.34	257.23	
TP055	BAHIA	Spain	medium		J-Temp	95.33	29.15	432.60	
TP056	BAIXET	Spain	long A		J-Temp	89.67	27.72	303.40	
TP057	BALILLA	Italy	round	1967	J-Temp	96.00	28.00	368.00	1
TP058	BALZARETTI	Italy	medium		J-Temp	92.00	17.88	330.87	
TP059	BARAGGIA	Italy	round	1957	J-Temp	86.33	24.98	294.22	
TP060	BEIRAO	Portugal	long A		J-Temp	78.00	21.92	264.22	
TP061	BELLE PATNA	USA	long B		J-Trop	103.17	18.17	392.14	
TP062	BENGAL	USA	long A		J-Temp	104.67	22.09	368.63	
TP063	BERTONE	Italy	long A	1930	J-Temp	77.17	19.32	254.63	
TP064	BIANCA	Italy	long A	2002	J-Temp	95.33	24.37	450.70	
TP065	BOMBILLA	Spain	medium		J-Temp	92.50	20.12	287.26	
TP066	BOMBON	Spain	medium	1975	J-Temp	109.67	19.90	351.63	
TP067	BONNI	Italy	long A		J-Temp	84.50	17.03	380.53	
TP068	BRAZOS	USA	long A		J-Trop	98.00	20.91	289.72	
TP069	BURMA	Italy	long A		J-Trop	87.67	22.12	351.93	
TP070	CALENDAL	France	long A		J-Temp	94.50	20.03	487.83	
TP071	CALMOCHI 101	USA	medium		J-Temp	90.67	22.26	293.47	
TP072	CAMPINO	Portugal	medium		J-Temp	90.33	21.04	391.32	
TP073	CAPATAZ	Spain	long A		J-Temp	99.00	19.47	224.52	
TP074	CARINA	Bulgary	round		J-Temp	98.83	19.44	385.08	
TP075	CARIOCA	Italy	long B	1975	J-Trop	86.67	24.38	293.97	
TP076	CARNISE	Italy	long A		J-Temp	97.00	24.16	321.94	1
TP077	CARRICO	Portugal	round		J-Temp	86.50	22.93	397.08	1
TP078	CASTELMOCHI	Italy	round		J-Temp	91.33	25.79	259.90	1
TP079	CHIPKA	Bulgary	round		J-Temp	86.83	24.13	493.12	1
TP080	CIGALON	France	medium		J-Temp	81.00	28.13	217.32	
TP081	CINIA 40	Chili			J-Temp	97.17	20.67	318.53	1
TP082	CLOT	Spain	medium		J-Temp	89.50	27.58	326.33	
TP083	COCODRIE	USA	long B	2004	J-Trop	98.83	18.82	248.13	
TP084	COLINA	Spain	round		J-Temp	93.50	28.38	364.00	
TP085	CORBETTA	Italy	medium	1954	J-Temp	83.50	26.71	527.22	1
TP086	CRIPTO	Italy		1978	J-Temp	85.83	23.22	368.27	
TP087	CT36	Colombia	long B		J-Temp	100.67	30.36	307.60	
TP088	CT58	Colombia	long A		J-Temp	93.33	18.19	164.62	1
TP089	DELLROSE	USA	long A		J-Trop	107.33	21.41	308.97	
TP090	DELMONT				J-Trop	106.67	19.46	280.34	

TP091	DIXIEBELLE	USA	long A		J-Trop	109.83	18.91	223.14	
TP092	DOURADAO				J-Trop	96.83	21.60	371.64	
TP093	DRAGO	Italy	long A	1990	J-Temp	88.67	28.58	411.69	1
TP094	DREW	USA	long B		J-Trop	108.00	21.29	351.29	
TP095	DUCATO	Italy	round	2011	J-Temp	90.17	26.12	286.15	
TP096	ERCOLE	Italy	long A		J-Temp	92.83	24.95	417.66	
TP097	ERMES	Italy	long B		J-Temp	95.00	21.33	300.70	
TP098	ESCARLATE	Portugal	round		J-Temp	81.17	23.44	223.91	
TP099	ESTRELA	Portugal	long A		J-Temp	84.67	25.06	238.22	
TP100	EUROPA	Italy	long A	1974	J-Temp	99.17	34.07	404.48	
TP101	EUROSE	Italy	long A		J-Temp	92.17	26.71	374.51	
TP102	FAMILIA 181	Portugal	long A		J-Temp	96.67	21.88	280.08	
TP103	FAST				J-Trop	89.22	26.56	237.00	
TP104	FIDJI	Philippine	long B	2001	J-temp	103.17	25.87	318.09	1
TP105	FLIPPER	Italy	long B	1997	J-Temp	89.17	26.34	306.59	1
TP106	FORTUNA	Italy	long A		J-Trop	110.67	16.48	311.94	
TP107	FRANCES	Spain	medium	2000	J-Temp	97.67	22.36	293.04	1
TP108	FULGENTE	Italy	medium		J-Temp	88.67	22.17	386.26	
TP109	GALILEO	Italy	long A	2002	J-Temp	89.33	23.18	385.86	1
TP110	GANGE	Italy	long B	1995	J-Trop	101.00	24.27	248.03	1
TP111	GARDE SADRI	Turkey	long A		J-Temp	93.50	27.53	391.43	
TP112	GIADA	Italy	long B		J-Trop	101.33	18.96	338.72	
TP113	GIOVANNI MARCHETTI	Italy	medium	1972	J-Temp	91.83	27.43	400.20	1
TP114	GITANO				J-Temp	86.83	29.05	313.06	
TP115	GIZA 177	Egypt	medium		J-Temp	96.50	22.32	308.15	
TP116	GLORIA				J-Temp	88.83	23.75	283.21	
TP117	GOOLARAH				J-Trop	115.67	17.88	200.95	
TP118	GRAAL	France	long B		J-Trop	85.83	24.99	299.24	
TP119	GRALDO	Italy	long B		J-Temp	91.17	18.99	312.26	
TP120	GREGGIO				J-Temp	92.00	21.02	383.21	
TP121	GREPPI	Italy	round	1908	J-Temp	104.83	23.49	384.83	1
TP122	GRITNA	Italy	long A		J-Temp	83.33	25.99	356.32	
TP123	GUADIAMAR	Spain	medium	1990	J-Temp	89.67	25.02	256.59	
TP124	GZ8367	Egypt			J-Temp	105.17	17.11	323.29	
TP125	HAREM	Portugal	long A		J-Temp	104.50	25.55	350.66	
TP126	HARRA	Australia	round		J-Temp	91.83	26.73	368.96	
TP127	HONDURAS	Spain	long A		J-Trop	115.29	17.73	231.18	
TP128	IAC32-52				J-Trop	109.17	16.66	479.07	
TP129	IBO 380-33	Portugal	long A		J-Temp	88.17	24.67	258.98	
TP130	IBO 400	Portugal	long A		J-Temp	97.67	27.58	438.99	
TP131	ITALMOCHI	Italy	medium	1996	J-Temp	81.33	22.43	252.95	
TP132	ITALPATNA 48	Italy	long A		J-Temp	100.17	26.49	416.62	
TP133	ITALPATNAxMILYANG	Portugal	long A		J-Temp	93.00	31.03	306.20	
TP134	JACINTO	USA	long A		J-Trop	103.17	17.36	305.16	
TP135	JEFFERSON	USA	long A	1996	J-Trop	97.33	24.17	323.73	
TP136	JUBILIENI	Bulgary	round		J-Temp	80.33	28.31	319.80	
TP137	KING	Italy	long B		J-Trop	94.83	22.27	332.32	
TP138	KRISTALLINO				J-Temp	87.97	28.21	501.83	1

TP139	KULON	Russia	long A		J-Temp	82.50	25.21	367.43	1
TP140	L201	USA	long B		J-Trop	96.00	17.15	307.66	
TP141	L202	USA	long B		J-Trop	100.33	20.98	303.54	1
TP142	L204	USA	long B		J-Trop	93.67	24.51	350.21	
TP143	L205	USA	long B		J-Trop	98.33	19.05	328.90	
TP144	LACASSINE	USA	long B		J-Trop	106.83	24.10	279.00	
TP145	LADY WRIGHT	USA	medium		J-Trop	111.33	18.87	386.92	1
TP146	LAGRUE	USA	long A		J-Trop	100.83	22.51	356.82	
TP147	LAMONE	Italy	long B	1999	J-Trop	94.50	23.09	291.53	
TP148	LENCINO	Italy	round	1930	J-Temp	90.50	20.20	300.05	
TP149	LIDO	Italy	medium	1976	J-Temp	92.33	22.39	272.09	1
TP150	LOMELLINO	Italy	medium	1982	J-Temp	79.83	24.40	305.30	
TP151	LORD	Italy	long A	1988	J-Temp	94.67	25.47	353.47	
TP152	LUCERO	Italy	round		J-Temp	101.67	22.59	342.73	
TP153	LUNA	USA	medium		J-Temp	100.17	24.07	351.80	
TP154	LUSITO IRRADIADO	Portugal	long A		J-Temp	92.17	22.12	297.31	
TP155	M202	USA	medium		J-Temp	91.83	25.08	335.35	
TP156	M203	USA	long A		J-Temp	95.00	23.75	376.41	1
TP157	M204	USA	long A		J-Temp	92.33	30.32	376.99	
TP158	M6	Italy	long A		J-Temp	90.67	26.69	330.40	
TP159	MAIORAL	Portugal	long A		J-Temp	99.33	21.82	348.08	
TP160	MANTOVA	Italy	long A	1933	J-Temp	93.00	25.84	401.44	
TP161	MARENY	Spain	long A		J-Temp	91.67	25.68	430.71	
TP162	MARTE	Italy	round	2000	J-Temp	93.67	30.26	332.84	
TP163	MAYBELLE	USA	long B		J-Trop	94.33	22.04	361.08	
TP164	MECO				J-Temp	92.33	29.70	415.18	
TP165	MEJANES	France	long B		J-Temp	86.00	22.12	304.99	
TP166	MELAS	Greece	long B		J-Temp	94.83	23.12	248.89	
TP167	MIARA	Italy	long B		J-Temp	82.33	25.90	177.63	
TP168	MILEV 21	Bulgary	round		J-Temp	89.33	22.04	447.78	
TP169	MOLO	Italy	long A		J-Trop	91.33	29.14	274.13	
TP170	MONTICELLI	Italy	medium	1967	J-Temp	92.67	22.82	446.11	
TP171	MUGA	Portugal	round		J-Temp	100.50	22.38	400.08	
TP172	MUSA				J-Temp	89.17	21.89	297.33	1
TP173	NANO	Italy	round		J-Temp	105.17	31.82	351.69	
TP174	NILO	Italy	long A		J-Temp	93.50	24.71	213.14	
TP175	NOVARA	Italy	medium	1933	J-Temp	83.33	19.46	305.00	
TP176	OLCENENGO	Italy	long A	1957	J-Temp	91.83	21.61	491.57	
TP177	ONICE				J-Temp	89.00	25.33	312.04	1
TP178	ORIGINARIO	Italy	round	1930	J-Temp	94.56	27.90	378.11	
TP179	ORIONE	Italy	long A		J-Temp	96.83	29.35	460.69	1
TP180	OSCARxSUWEON	Portugal	long A		J-Temp	95.67	21.88	416.00	
TP181	OSTIGLIA	Italy	round	1923	J-Temp	88.00	19.36	289.14	
TP182	OTA	Portugal	long A		J-Temp	103.17	18.32	412.54	
TP183	P6	Italy	medium		J-Temp	89.67	21.12	328.05	
TP184	PADANO	Italy	long A		J-Temp	96.17	27.79	434.67	
TP185	PANDA	Italy		1988	J-Trop	93.00	24.73	382.88	
TP186	PEGONIL	Spain	medium		J-Temp	97.50	23.99	487.45	

TP187	PELDE	Australia			J-Temp	79.67	23.46	168.40	
TP188	PIEMONTE	Italy	long A	1983	J-Temp	93.50	29.06	467.27	
TP189	PIERINA MARCHETTI	Italy	long A		J-Temp	93.00	25.22	544.97	
TP190	PLOVDIV 22	Bulgary	long A		J-Temp	81.17	25.12	338.32	
TP191	PLOVDIV 24	Bulgary	round		J-Temp	90.19	30.26	388.40	
TP192	PLUS	Italy	long B		J-Trop	105.83	20.82	328.44	
TP193	PREVER	Italy	long B	1989	J-Temp	84.83	19.99	345.67	
TP194	PROMETEO	Italy	medium	1990	J-Temp	87.83	20.66	386.72	
TP195	PUNTAL	Spain	long B	1991	J-Trop	103.83	20.10	396.72	
TP196	RANGHINO	Italy	round		J-Temp	83.50	22.47	247.38	
TP197	RAZZA 77	Italy	medium		J-Temp	88.33	25.86	370.34	
TP198	REDI	Italy	long A	1967	J-Temp	96.00	23.77	421.35	1
TP199	REXMONT	USA	long B		J-Trop	105.50	22.48	308.55	
TP200	RIBE	Italy	long A	1967	J-Temp	94.67	30.04	339.84	1
TP201	RINALDO BERSANI	Italy	long A		J-Temp	96.67	22.62	399.64	
TP202	RINGO	Italy	long A	1972	J-Temp	97.50	27.12	471.18	
TP203	RIZZOTTO 51 1	Italy	long A		J-Temp	98.33	23.35	497.59	
TP204	ROBBIO SEL1	Italy	long A		J-Temp	94.33	22.33	342.00	
TP205	RODEO	Italy	long A	2002	J-Temp	79.33	24.32	265.32	
TP206	RODINA	Bulgary	round		J-Temp	92.50	18.92	479.80	
TP207	ROMA	Italy	long A	1967	J-Temp	97.33	20.67	530.46	
TP208	RONALDO	Italy	long A		J-Temp	93.33	24.78	377.14	1
TP209	RONCAROLO				J-Temp	99.33	22.99	503.40	
TP210	RONCOLO	Italy	medium		J-Temp	94.50	26.65	460.89	
TP211	ROTUNDUS	Hungary	long A		J-Temp	80.17	22.65	225.00	
TP212	ROXANI	Greece	long A		J-Temp	101.67	21.90	426.48	
TP213	RPC 12	China	round		J-Temp	81.67	24.12	219.03	1
TP214	RUBI	Portugal			J-Temp	96.50	28.04	341.08	
TP215	RUBINO	Italy	round	1978	J-Temp	96.83	24.95	494.54	
TP216	RUSSO	Italy			J-Temp	73.50	20.13	197.95	
TP217	S101	USA	medium		J-Temp	88.83	24.83	313.95	1
TP218	SAEDINENIE	Bulgary	long A		J-Temp	80.50	22.85	420.40	
TP219	SAFARI	Portugal	long A		J-Temp	95.83	26.94	413.12	
TP220	SAGRES	Portugal	long A		J-Temp	102.00	20.94	340.56	
TP221	SAKHA 102	Egypt	medium		J-Temp	99.33	21.63	309.92	
TP222	SAKHA 103	Egypt	round		J-Temp	99.50	23.27	327.95	
TP223	SALOIO	Portugal	long B		J-Temp	88.33	22.83	263.52	
TP224	SALVO	Italy	long B	2008	J-Trop	92.17	20.28	308.80	1
TP225	SAMBA	Italy	long A		J-Trop	89.17	26.73	325.36	
TP226	SANDOCA	Portugal	long B		J-Temp	101.00	22.30	350.28	
TP227	SANDORA	Hungary	long A		J-Temp	74.33	21.13	162.66	
TP228	SANTANDREA	Italy	long A	1974	J-Temp	91.17	21.82	426.91	
TP229	SANTERNO	Italy	long B	1998	J-Temp	99.17	18.59	291.84	
TP230	SATURNO	Italy	long B		J-Trop	89.67	26.38	246.46	1
TP231	SAVIO	Italy	long A	1995	J-Temp	88.50	26.84	289.26	
TP232	SCUDO	Italy	long B		J-Trop	93.33	19.48	333.98	
TP233	SELN 244A620	Australia	medium		J-Temp	96.00	23.99	328.78	
TP234	SENATORE NOVELLI	Italy	long A		J-Temp	92.33	19.44	315.21	1

TP235	SENIA	Spain	medium	1986	J-Temp	94.83	26.50	368.78	1
TP236	SEQUIAL	Spain	medium		J-Temp	93.17	26.82	307.78	
TP237	SESIA	Italy	long A		J-Temp	93.17	25.64	305.01	
TP238	SESIAMOCHI	Italy	long A		J-Temp	90.00	24.30	439.40	1
TP239	SETANTUNO	Portugal	round		J-Temp	95.00	23.07	652.79	1
TP240	SFERA				J-Temp	89.50	24.26	225.78	1
TP241	SHSS 381	Spain	long A		J-Temp	96.00	26.77	432.23	1
TP242	SHSS 53	Spain	long A		J-Temp	96.17	26.90	363.70	
TP243	SILLA	Italy	long A	1973	J-Temp	83.83	24.67	338.02	
TP244	SIRIO	Italy	long A		J-Trop	85.17	26.43	336.75	1
TP245	SLAVA	Bulgary	medium		J-Temp	91.50	23.22	302.95	
TP246	SMERALDO	Italy	long A	1982	J-Temp	89.33	29.77	400.42	
TP247	SOURE	Portugal	long A		J-Temp	94.50	20.17	303.65	
TP248	SPRINT	Italy	long B	2002	J-Trop	88.33	25.62	270.30	
TP249	SR 113	Spain	long A		J-Temp	92.50	23.59	385.38	1
TP250	STRELLA	Italy	long A	1981	J-Temp	91.83	24.38	338.97	
TP251	SUPER	Portugal			J-Temp	92.67	23.99	352.16	
TP252	T757	India			J-Temp	91.17	27.14	423.91	
TP253	TAICHUNG 65	Thailand			J-Temp	94.00	27.95	341.79	
TP254	TEXMONT	USA	long A		J-Trop	98.33	26.89	311.49	
TP255	THAIBONNET	Italy	long B	1992	J-Trop	101.83	17.91	275.32	1
TP256	THAIPERLA				J-Temp	92.00	25.94	370.25	1
TP257	TITANIO	Italy			J-Temp	77.33	26.36	360.47	1
TP258	TOPAZIO	Italy	medium		J-Temp	80.83	22.74	343.45	
TP259	TORIO	Portugal	long A		J-Temp	91.33	23.95	417.37	
TP260	ULISSE	Italy	long A		J-Temp	93.17	30.12	360.36	
TP261	ULLAL	Spain	round	1998	J-Temp	91.17	33.12	379.08	
TP262	UPLA 32	Argentina	long B		J-Trop	96.50	22.36	218.18	
TP263	UPLA 63	Argentina	long B		J-Trop	101.83	16.86	295.25	
TP264	UPLA 64	Argentina	long B		J-Trop	94.33	19.53	278.03	
TP265	UPLA 66	Argentina	long B		J-Trop	93.67	20.42	277.94	
TP266	UPLA 68	Argentina	long B		J-Trop	102.00	18.91	304.48	
TP267	UPLA 75	Argentina	long B		J-Trop	101.67	21.23	286.16	
TP268	UPLA 77	Argentina	long B		J-Trop	98.33	22.77	244.43	
TP269	UPLA 80	Argentina	long B		J-Trop	94.50	22.13	348.41	
TP270	UPLA 91	Argentina	long B		J-Trop	101.05	21.41	320.25	
TP271	VALTEJO	Portugal	round		J-Temp	96.50	21.62	384.50	
TP272	VELA	Italy	long A		J-Temp	96.33	21.31	255.84	
TP273	VENERE	Italy	long B	1997	J-Temp	84.67	20.32	209.37	
TP274	VENERIA	Italy	long A	1978	J-Temp	92.17	25.14	456.15	1
TP275	VIALE	Italy	long A		J-Temp	90.33	26.47	316.98	
TP276	VIALONE 190	Italy	medium		J-Temp	91.17	21.37	343.68	1
TP277	VIALONE NERO				J-Temp	97.50	18.56	422.61	1
TP278	VICTORIA	Argentina	round		J-Temp	89.33	19.40	268.07	1
TP279	VIRGO				J-Temp	88.00	27.42	465.88	
TP280	VULCANO				J-Temp	97.50	23.05	499.76	
TP281	XIANGHU2				J-temp	93.50	19.82	277.02	
TP282	YRM 6 2	Australia	medium		J-Temp	94.33	27.33	412.06	

TP283	ZENA	Italy	long B	1994	J-Trop	91.33	20.84	343.71		1
TP284	ZENITH	USA	medium		J-Trop	116.00	15.59	324.08		

Supplementary Table 2: List of the 97 F5-F7 lines of the progeny population and their phenotype for days to flowering (FL), nitrogen balance index (NI) and 100 panicles weight (PW).

Genotype	Crosses	FL (days)	NI	PW (g)
PF043	Aiace / Perla	97.83	15.42	184.71
PF048	Apollo / Volano	96.50	15.64	272.91
PF031	Apollo/Selenio//Apollo	92.33	17.14	216.46
PF081	Apollo/Selenio//Apollo	94.00	16.76	255.47
PF057	Asia / 2*Selenio	94.83	20.34	316.05
PF058	Asia / 2*Selenio	94.83	19.00	195.4
PF059	Asia / 2*Selenio	107.00	20.86	210.07
PF085	Asia / Centauro	97.00	17.23	182.2
PF087	Asia / Centauro	96.33	17.11	243.27
PF030	Augusto / Gigante Vercelli	80.17	22.66	281.54
PF069	Augusto / Handao 297	94.67	20.38	173.15
PF070	Augusto / Handao 297	95.33	19.95	227.75
PF042	Baldo / Handao 297	91.50	19.29	398.7
PF052	Baldo / Handao 297	85.50	22.22	303.79
PF053	Baldo / Handao 297	90.67	18.91	285.46
PF055	Baldo / Handao 297	90.17	17.72	284.33
PF019	Baldo / Opale	90.67	17.58	292.24
PF020	Baldo / Opale	91.67	18.93	380.09
PF021	Baldo / Opale	96.17	17.77	328.84
PF022	Baldo / Opale	90.33	18.78	274.58
PF023	Baldo / Opale	83.83	17.45	278.17
PF024	Baldo / Opale	90.83	18.55	286.5
PF015	Carmen / Creso	95.00	18.17	323.01
PF016	Carmen / Creso	93.50	17.71	301.36
PF017	Carmen / Creso	95.50	16.32	325.19
PF026	Carmen / Loto	89.26	17.28	276.74
PF027	Carmen / Loto	88.83	19.63	182.44
PF068	Centauro / Dimitra	89.00	20.94	259.01
PF051	Centauro / Koral	83.33	21.77	289.97
PF071	Centauro / Koral	100.17	23.66	286.79
PF072	Centauro / Koral	86.33	20.91	254.45
PF073	Centauro / Koral	99.83	23.96	280.09
PF047	Creso / Apollo	91.50	18.25	259.26
PF001	Delfino / 2*Centauro	88.67	18.84	248.21
PF002	Delfino / 2*Centauro	92.00	19.18	256.57
PF067	Delfino / Centauro	95.00	21.56	342.31
PF100	Delfino / Selenio	84.00	19.12	196.06
PF084	Eurosis / Gladio	88.67	15.75	233.59
PF004	Eurosis / Handao 11	88.00	18.17	303.74
PF005	Eurosis / Handao 11	86.00	12.21	228.03
PF006	Eurosis / Handao 11	90.93	18.91	320.01
PF007	Eurosis / Handao 11	97.67	19.17	293.16
PF008	Eurosis / Handao 11	102.50	18.19	308.41

PF009	Eurosis / Handao 11	96.83	15.53	245.35
PF010	Eurosis / Handao 11	102.50	14.59	240.41
PF011	Eurosis / Handao 11	95.00	17.18	299.83
PF012	Eurosis / Handao 11	92.83	17.37	363.48
PF013	Eurosis / Handao 11	101.50	17.72	298.86
PF028	Eurosis / Handao 11	83.67	18.31	240.69
PF032	Eurosis / Handao 11	108.83	19.14	256.69
PF033	Eurosis / Handao 11	90.50	25.13	247.38
PF034	Eurosis / Handao 11	96.33	16.38	240.44
PF035	Eurosis / Handao 11	88.83	18.90	287.98
PF036	Eurosis / Handao 11	92.83	20.43	248.68
PF037	Eurosis / Handao 11	91.00	20.33	241.5
PF038	Eurosis / Handao 11	91.33	14.71	266.5
PF039	Eurosis / Handao 11	98.00	15.85	304.32
PF046	Eurosis / Handao 11	87.67	15.57	283.06
PF082	Fragrance / Karnak	107.50	21.35	219.11
PF091	Giano / Loto	89.50	18.37	155.66
PF025	Giano / Vialone Nano	90.83	17.84	391.12
PF054	Giano / Vialone Nano	86.33	15.41	222.66
PF092	Giano / Vialone Nano	89.83	18.95	395.66
PF093	Giano / Vialone Nano	90.50	19.66	397.67
PF094	Giano / Vialone Nano	94.17	18.59	268.45
PF095	Giano / Vialone Nano	93.33	20.08	210.72
PF096	Giano / Vialone Nano	90.17	16.89	243.39
PF097	Giano / Vialone Nano	91.33	19.50	267.96
PF098	Giano / Vialone Nano	94.17	20.64	219.17
PF060	Gladio / Eurosis /Gladio	90.00	16.62	384.51
PF061	Gladio / Opale	96.50	16.63	276.44
PF062	Gladio / Opale	89.33	17.80	417.21
PF063	Gladio / Opale	88.83	14.40	319.45
PF064	Gladio / Opale	98.17	14.49	219.05
PF065	Gladio / Opale	92.67	17.17	341.53
PF066	Gladio / Opale	97.17	18.15	292.72
PF049	Handao 297 / Luxor	89.33	18.73	322.09
PF050	Handao 297 / Luxor	98.17	20.34	275.73
PF040	Karnak / 2*Giano	94.67	18.17	290.98
PF041	Karnak / 2*Giano	96.00	20.00	304.77
PF076	Karnak / Delfino	95.67	18.12	354.78
PF077	Karnak / Delfino	95.33	19.48	349.45
PF078	Karnak / Delfino	95.67	18.57	337.78
PF075	Karnak / Giano	96.33	17.23	292.19
PF088	Karnak / Giano	96.50	17.36	207.7
PF056	Loto / Karnak	93.33	18.88	288.34
PF044	Maratelli / Carmen	84.17	20.52	269.72
PF045	Maratelli / Carmen	85.67	20.44	273.15
PF083	Pecos / Delfino	89.50	17.10	322.59
PF014	Pecos / Gladio	92.00	19.43	221.21
PF099	Pecos / Gladio	101.83	18.14	234.23

PF089	SIS R215 / Carnaroli	91.17	18.32	239.18
PF090	SIS R215 / Carnaroli	87.17	19.45	235.35
PF079	SIS R215 / Loto	90.67	19.08	337.12
PF080	SIS R215 / Loto	91.42	16.22	305.25
PF029	Tejo / Aiace	90.50	19.30	219.68
PF074	Tejo / Centauro	93.83	23.48	330.89

Supplementary Table 3: Variability of marker density and frequency of minor allele along the 12 chromosomes of rice, within the reference and the progeny populations

			Chr1	Chr2	Chr3	Chr4	Chr5	Chr6	Chr7	Chr8	Chr9	Chr10	Chr11	Chr12	All
**Distribution of distance (bp) between two adjacent SNP loci**		**Size ()**	43 154 269	35 770 733	36 303 800	35 495 001	29 863 328	31 092 718	29 650 697	28 424 033	22 791 537	23 065 658	29 015 845	27377100	27 410 737
		**Number**	5183	3719	2953	3553	2576	3116	3199	3937	1947	4378	6128	2997	43 686
		**density**	120	86	68	82	60	72	74	91	45	101	142	69	1012
		**Minimum**	1	1	1	1	1	1	3	1	2	1	1	1	1
		**1st Quartile**	103	71	74	46	79	63.5	60	57	67	43	36	46	46
		**Median**	1834	1651.5	1 756	1 096	1 919	1601	1 329	1 371	2 049	531	299	851	1 486
		**3rd Quartile**	7591	9367	10720	8428	9186	9243	7951	7696	11592	5328	4255	8159	9274
		**Maximum**	432999	403965	347 318	643 880	458 043	9242.5	399 519	289 104	344 748	195 190	273 928	340246	643 880
**Distribution of the minor allele frequency (MAF %)**	**Reference population**	**Minimum**	0.0	0.0	0.0	0.0	0.0	0.0	0.0	0.0	0.4	0.0	0.0	0.0	0.0
		**1st Quartile**	3.9	3.9	4.2	6.4	7.1	5.0	6.3	4.2	4.6	5.6	3.6	10.2	4.6
		**Median**	12.7	10.2	10.6	26.1	31.5	17.7	25.4	17.0	20.8	20.4	12.4	24.4	17.7
		**3rd Quartile**	24.7	31.1	26.5	42.0	43.5	33.1	37.0	34.6	33.5	35.3	28.5	39.0	34.6
		**Maximum**	50.0	50.0	49.8	50.0	50.0	49.8	50.0	49.8	50.0	50.0	50.0	49.3	50.0
	**Progeny population**	**Minimum**	0.0	0.0	0.0	0.0	0.0	0.0	0.0	0.0	0.0	0.0	0.0	0.0	0.0
		**1st Quartile**	8.0	3.1	6.1	7.1	11.2	6.1	7.1	4.1	5.1	6.1	8.2	12.2	7.1
		**Median**	14.3	14.3	13.3	18.4	30.6	15.3	18.4	18.4	20.4	15.8	16.3	27.6	16.3
		**3rd Quartile**	23.5	25.5	26.5	35.7	41.8	30.6	31.6	38.8	30.6	31.6	26.5	38.8	31.6
		**Maximum**	50.0	50.0	50.0	50.0	50.0	50.0	50.0	50.0	50.0	50.0	50.0	50.0	50.0

Supplementary Table 4: Variability of decay of pairwise linkage disequilibrium with distance be

	Chr	0-25	25-50	50-75	75-100	100-125	125-150	150-175
Reference population	1	0.614	0.446	0.422	0.395	0.376	0.372	0.356
	2	0.613	0.444	0.391	0.373	0.357	0.331	0.328
	3	0.693	0.505	0.493	0.459	0.432	0.443	0.402
	4	0.613	0.402	0.372	0.366	0.316	0.335	0.312
	5	0.763	0.631	0.600	0.580	0.559	0.542	0.537
	6	0.646	0.448	0.409	0.374	0.340	0.327	0.324
	7	0.685	0.504	0.479	0.446	0.444	0.420	0.416
	8	0.607	0.400	0.361	0.347	0.326	0.314	0.291
	9	0.675	0.449	0.423	0.393	0.399	0.365	0.343
	10	0.526	0.367	0.351	0.334	0.316	0.329	0.304
	11	0.482	0.296	0.256	0.243	0.235	0.233	0.224
	12	0.621	0.457	0.414	0.396	0.386	0.381	0.389
	Mean	0.628	0.446	0.414	0.392	0.374	0.366	0.352
	Stdev	0.075	0.082	0.085	0.081	0.081	0.077	0.079
Progeny population	1	0.649	0.498	0.478	0.453	0.435	0.427	0.411
	2	0.671	0.520	0.468	0.458	0.435	0.410	0.403
	3	0.722	0.549	0.545	0.514	0.487	0.494	0.469
	4	0.655	0.449	0.421	0.406	0.360	0.366	0.347
	5	0.771	0.629	0.590	0.572	0.551	0.536	0.520
	6	0.679	0.495	0.458	0.423	0.397	0.385	0.392
	7	0.723	0.554	0.524	0.487	0.496	0.470	0.460
	8	0.650	0.448	0.418	0.399	0.379	0.364	0.350
	9	0.697	0.498	0.467	0.428	0.432	0.413	0.400
	10	0.570	0.416	0.395	0.382	0.363	0.377	0.347
	11	0.513	0.323	0.277	0.265	0.254	0.244	0.238
	12	0.643	0.486	0.454	0.431	0.422	0.408	0.408
	Mean	0.662	0.489	0.458	0.435	0.417	0.408	0.395
	Stdev	0.069	0.077	0.080	0.075	0.077	0.074	0.072

etween markers among the 12 chromosomes within the Reference and the Progeny population

175-200	200-225	225-250	250-300	300-350	350-400	400-450	450-500	500-600
0.334	0.318	0.320	0.315	0.295	0.285	0.270	0.252	0.243
0.329	0.312	0.282	0.280	0.252	0.225	0.205	0.278	0.173
0.391	0.394	0.374	0.349	0.345	0.304	0.304	0.416	0.294
0.299	0.292	0.289	0.291	0.282	0.279	0.246	0.251	0.211
0.517	0.499	0.474	0.464	0.449	0.426	0.420	0.347	0.420
0.302	0.287	0.274	0.257	0.237	0.212	0.190	0.191	0.179
0.422	0.403	0.426	0.417	0.399	0.394	0.392	0.394	0.364
0.270	0.266	0.257	0.258	0.238	0.221	0.213	0.208	0.206
0.345	0.333	0.335	0.307	0.277	0.292	0.264	0.246	0.231
0.292	0.266	0.275	0.264	0.260	0.258	0.250	0.228	0.222
0.215	0.209	0.198	0.199	0.184	0.171	0.174	0.161	0.159
0.382	0.365	0.343	0.339	0.307	0.317	0.319	0.289	0.281
0.342	0.329	0.320	0.312	0.294	0.282	0.271	0.272	0.249
0.079	0.077	0.076	0.073	0.074	0.074	0.077	0.079	0.079
0.384	0.373	0.370	0.369	0.349	0.336	0.320	0.303	0.286
0.388	0.361	0.346	0.352	0.344	0.297	0.278	0.273	0.244
0.462	0.455	0.443	0.416	0.402	0.373	0.366	0.357	0.357
0.332	0.329	0.330	0.329	0.322	0.315	0.282	0.267	0.252
0.503	0.490	0.467	0.472	0.458	0.433	0.416	0.424	0.408
0.358	0.342	0.350	0.326	0.301	0.278	0.264	0.248	0.238
0.466	0.453	0.466	0.447	0.446	0.435	0.429	0.450	0.391
0.321	0.323	0.314	0.309	0.282	0.262	0.251	0.239	0.244
0.378	0.357	0.358	0.356	0.329	0.331	0.298	0.274	0.260
0.334	0.308	0.314	0.299	0.298	0.300	0.291	0.273	0.260
0.232	0.226	0.214	0.217	0.201	0.188	0.183	0.173	0.169
0.404	0.394	0.371	0.358	0.335	0.348	0.347	0.319	0.311
0.380	0.368	0.362	0.354	0.339	0.325	0.310	0.300	0.285
0.074	0.073	0.072	0.069	0.071	0.070	0.070	0.078	0.070

600-700	700-800	800-900	900-1000	1000-1500	1500-2000	2000-2500	2500-3000	3000-3500
0.235	0.222	0.208	0.199	0.194	0.168	0.144	0.117	0.095
0.172	0.150	0.146	0.143	0.154	0.141	0.112	0.093	0.069
0.266	0.241	0.199	0.176	0.154	0.120	0.102	0.085	0.068
0.216	0.207	0.174	0.167	0.160	0.133	0.124	0.116	0.102
0.396	0.365	0.302	0.275	0.223	0.148	0.119	0.101	0.100
0.166	0.171	0.138	0.134	0.149	0.118	0.090	0.077	0.058
0.336	0.306	0.287	0.269	0.224	0.178	0.146	0.120	0.099
0.196	0.178	0.178	0.175	0.174	0.155	0.135	0.125	0.113
0.221	0.201	0.179	0.169	0.165	0.165	0.141	0.115	0.115
0.210	0.201	0.185	0.177	0.171	0.149	0.133	0.122	0.118
0.151	0.138	0.127	0.116	0.114	0.098	0.091	0.082	0.079
0.282	0.264	0.244	0.234	0.194	0.159	0.137	0.128	0.105
0.237	0.220	0.197	0.186	0.173	0.144	0.123	0.107	0.093
0.072	0.066	0.056	0.050	0.032	0.023	0.020	0.018	0.020
0.271	0.250	0.241	0.229	0.214	0.175	0.149	0.124	0.105
0.245	0.243	0.240	0.218	0.183	0.136	0.123	0.105	0.088
0.333	0.313	0.284	0.260	0.213	0.170	0.152	0.116	0.106
0.261	0.252	0.222	0.200	0.187	0.161	0.157	0.148	0.126
0.372	0.344	0.298	0.272	0.201	0.127	0.112	0.109	0.106
0.214	0.203	0.179	0.165	0.149	0.121	0.099	0.085	0.063
0.342	0.312	0.311	0.317	0.243	0.205	0.167	0.133	0.104
0.234	0.215	0.212	0.220	0.194	0.181	0.166	0.151	0.138
0.260	0.244	0.227	0.212	0.208	0.211	0.172	0.147	0.146
0.249	0.232	0.222	0.214	0.194	0.176	0.173	0.159	0.151
0.158	0.145	0.142	0.140	0.116	0.100	0.095	0.087	0.085
0.314	0.289	0.265	0.256	0.228	0.193	0.162	0.163	0.156
0.271	0.254	0.237	0.225	0.194	0.163	0.144	0.127	0.114
0.060	0.055	0.048	0.047	0.034	0.035	0.029	0.027	0.029

3500-4000	4000-4500	4500-5000	>5000
0.086	0.074	0.066	0.049564
0.060	0.053	0.050	0.027973
0.074	0.069	0.066	0.045006
0.106	0.085	0.070	0.044266
0.118	0.105	0.089	0.071887
0.056	0.045	0.046	0.029196
0.087	0.077	0.065	0.031771
0.106	0.088	0.076	0.032494
0.100	0.076	0.070	0.041614
0.104	0.089	0.088	0.040883
0.081	0.077	0.073	0.027947
0.096	0.100	0.091	0.047847
0.089	0.078	0.071	0.041
0.019	0.017	0.014	0.013
0.088	0.081	0.071	0.062787
0.067	0.060	0.058	0.033476
0.107	0.092	0.077	0.029455
0.118	0.086	0.060	0.036513
0.106	0.084	0.064	0.03203
0.053	0.051	0.054	0.036898
0.097	0.085	0.069	0.040035
0.130	0.107	0.094	0.044142
0.139	0.101	0.093	0.040212
0.136	0.122	0.121	0.051289
0.087	0.084	0.079	0.041198
0.136	0.135	0.122	0.059044
0.105	0.091	0.080	0.042
0.028	0.024	0.023	0.010

Supplementary Table 5: Average accuracy of cross validation for days to flowering (FL), nitrogen bal

LD (r^2)	MAF (%)	FL BayesB	FL GBLUP	FL RKHS	NI BayesB	NI GBLUP	NI RKHS
0.25	5	0.64	0.64	0.64	0.43	0.43	0.43
0.25	10	0.62	0.61	0.61	0.43	0.43	0.44
0.25	20	0.57	0.57	0.58	0.42	0.43	0.43
0.36	5	0.63	0.63	0.63	0.47	0.46	0.47
0.36	10	0.63	0.63	0.63	0.46	0.46	0.47
0.36	20	0.61	0.61	0.62	0.44	0.44	0.44
0.49	5	0.65	0.62	0.65	0.53	0.52	0.53
0.49	10	0.63	0.63	0.63	0.51	0.48	0.52
0.49	20	0.61	0.61	0.62	0.51	0.51	0.51
0.64	5	0.64	0.61	0.64	0.57	0.54	0.57
0.64	10	0.64	0.62	0.64	0.55	0.52	0.55
0.64	20	0.63	0.63	0.64	0.55	0.54	0.55
0.81	5	0.65	0.61	0.65	0.56	0.53	0.55
0.81	10	0.65	0.60	0.65	0.54	0.51	0.54
0.81	20	0.64	0.64	0.65	0.54	0.51	0.54
0.98	5	0.65	0.61	0.65	0.55	0.51	0.55
0.98	10	0.64	0.59	0.65	0.54	0.49	0.54
0.98	20	0.63	0.60	0.64	0.54	0.48	0.54
1	5	0.65	0.59	0.65	0.54	0.50	0.54
1	10	0.63	0.58	0.64	0.53	0.48	0.53
1	20	0.63	0.59	0.64	0.53	0.46	0.53

The confidance interval fro each average accuracy is +/- 0.01

ance index (NI) and 100 panicle weight (PW), obtain with three statistical methods, Bayes B, GBLUP

	PW	
**BayesB**	**GBLUP**	**RKHS**
0.56	0.56	0.57
0.53	0.54	0.54
0.53	0.54	0.54
0.59	0.59	0.59
0.56	0.56	0.57
0.56	0.56	0.57
0.61	0.61	0.61
0.60	0.60	0.61
0.60	0.60	0.60
0.61	0.60	0.61
0.60	0.60	0.60
0.62	0.62	0.62
0.60	0.60	0.60
0.61	0.61	0.62
0.62	0.62	0.62
0.59	0.58	0.59
0.58	0.57	0.58
0.58	0.58	0.58
0.57	0.57	0.58
0.59	0.59	0.60
0.57	0.57	0.58

' and RKHS

Supplementary Table 6: Accuracy of genomic prediction of progeny phenotype for days to flc panicles weight (PW), obtain with three statistical methods, Bayes B, GBLUP and RKHS, under training and the progeny set.

			FL			NI	
Scenario	r^2	MAF (%)	BayesB	GBLUP	RKHS	BayesB	GBLUP
S1	0.25	5	0.25	0.27	0.26	0.19	0.20
	0.36	5	0.28	0.30	0.28	0.24	0.24
	0.49	5	0.28	0.32	0.30	0.18	0.19
	0.64	5	0.27	0.25	0.30	0.17	0.16
	0.81	5	0.28	0.26	0.30	0.18	0.18
	0.98	5	0.27	0.24	0.28	0.20	0.19
	1a	5	0.26	0.23	0.29	0.20	0.19
	1b	2.5	0.25	0.23	0.28	0.18	0.18
S2	0.25	5	0.35	0.34	0.35	0.23	0.23
	0.36	5	0.37	0.37	0.39	0.30	0.31
	0.49	5	0.46	0.44	0.45	0.37	0.36
	0.64	5	0.40	0.41	0.42	0.40	0.38
	0.81	5	0.41	0.40	0.42	0.40	0.41
	0.98	5	0.33	0.32	0.35	0.33	0.31
	1a	5	0.31	0.27	0.31	0.24	0.22
	1b	2.5	0.31	0.28	0.32	0.23	0.22
S3	0.25	5	0.29	0.32	0.31	0.27	0.28
	0.36	5	0.33	0.32	0.29	0.34	0.36
	0.49	5	0.40	0.39	0.37	0.39	0.36
	0.64	5	0.38	0.35	0.37	0.42	0.38
	0.81	5	0.39	0.36	0.39	0.42	0.40
	0.98	5	0.35	0.32	0.34	0.38	0.40
	1a	5	0.31	0.29	0.33	0.34	0.37
	1b	2.5	0.31	0.28	0.32	0.33	0.35
S4	0.25	5	0.31	0.31	0.30	0.30	0.31
	0.36	5	0.33	0.32	0.32	0.44	0.42
	0.49	5	0.42	0.34	0.40	0.52	0.49
	0.64	5	0.45	0.38	0.43	0.48	0.47
	0.81	5	0.41	0.35	0.40	0.49	0.48
	0.98	5	0.36	0.37	0.36	0.46	0.46
	1a	5	0.31	0.35	0.30	0.45	0.45
	1b	2.5	0.27	0.31	0.28	0.42	0.42
S5	0.25	5	0.39	0.39	0.38	0.18	0.20
	0.36	5	0.40	0.39	0.38	0.34	0.32
	0.49	5	0.48	0.43	0.47	0.43	0.37
	0.64	5	0.51	0.49	0.51	0.43	0.41
	0.81	5	0.46	0.44	0.46	0.44	0.42
	0.98	5	0.39	0.43	0.40	0.41	0.40
	1a	5	0.30	0.40	0.32	0.38	0.38
	1b	2.5	0.28	0.34	0.29	0.38	0.36
	0.25	5	0.19 ± 0.01	0.19 ± 0.01	0.19 ± 0.01	0.12 ± 0.01	0.14 ± 0.01
	0.36	5	0.20 ± 0.01	0.19 ± 0.01	0.20 ± 0.01	0.18 ± 0.01	0.21 ± 0.01

S6	0.49	5	0.21 ± 0.01	0.19 ± 0.01	0.20 ± 0.01	0.22 ± 0.01	0.22 ± 0.01
	0.64	5	0.21 ± 0.01	0.22 ± 0.01	0.22 ± 0.01	0.24 ± 0.01	0.24 ± 0.01
	0.81	5	0.19 ± 0.01	0.19 ± 0.01	0.18 ± 0.01	0.24 ± 0.01	0.23 ± 0.01
	0.98	5	0.12 ± 0.01	0.12 ± 0.01	0.11 ± 0.01	0.21 ± 0.02	0.21 ± 0.02
	1a	5	0.06 ± 0.01	0.07 ± 0.01	0.06 ± 0.01	0.20 ± 0.02	0.18 ± 0.02
	1b	2.5	0.06 ± 0.01	0.07 ± 0.01	0.05 ± 0.01	0.19 ± 0.02	0.19 ± 0.02

1-a and 1-b, represent incidence matrixes without selection on r^2, but filtered with MAF > 5 %

owering (FL), nitrogen balance index (NI) and 100
six scenarios of relatedness between the

	PW		
RKHS	BayesB	GBLUP	RKHS
0.09	0.48	0.49	0.48
0.13	0.50	0.52	0.49
0.13	0.53	0.54	0.50
0.12	0.50	0.50	0.47
0.15	0.46	0.48	0.47
0.23	0.45	0.49	0.45
0.26	0.44	0.51	0.42
0.23	0.46	0.52	0.43
0.24	0.28	0.30	0.29
0.32	0.30	0.30	0.28
0.36	0.33	0.29	0.27
0.39	0.31	0.31	0.28
0.38	0.29	0.32	0.27
0.34	0.28	0.29	0.22
0.27	0.31	0.33	0.28
0.28	0.36	0.37	0.34
0.28	0.45	0.44	0.44
0.36	0.46	0.43	0.43
0.39	0.48	0.46	0.44
0.42	0.45	0.45	0.44
0.41	0.42	0.44	0.44
0.38	0.38	0.41	0.40
0.34	0.41	0.43	0.41
0.34	0.42	0.45	0.44
0.31	0.35	0.34	0.31
0.44	0.36	0.35	0.44
0.50	0.39	0.39	0.50
0.48	0.39	0.39	0.48
0.49	0.40	0.39	0.49
0.46	0.38	0.38	0.46
0.45	0.37	0.38	0.45
0.43	0.38	0.39	0.43
0.20	0.18	0.18	0.17
0.34	0.21	0.19	0.19
0.42	0.27	0.26	0.26
0.43	0.28	0.28	0.28
0.45	0.30	0.30	0.30
0.41	0.29	0.29	0.29
0.39	0.31	0.30	0.30
0.38	0.31	0.31	0.31
0.13 ± 0.01	0.21 ± 0.01	0.24 ± 0.01	0.23 ± 0.01
0.20 ± 0.01	0.24 ± 0.01	0.28 ± 0.01	0.27 ± 0.01

0.23 ± 0.01	0.28 ± 0.01	0.33 ± 0.01	0.31 ± 0.01
0.26 ± 0.01	0.30 ± 0.01	0.35 ± 0.01	0.33 ± 0.01
0.26 ± 0.01	0.32 ± 0.01	0.36 ± 0.01	0.35 ± 0.01
0.24 ± 0.01	0.30 ± 0.01	0.34 ± 0.01	0.33 ± 0.01
0.23 ± 0.02	0.30 ± 0.01	0.34 ± 0.01	0.33 ± 0.01
0.23 ± 0.02	0.29 ± 0.01	0.33 ± 0.01	0.32 ± 0.01

[illegible] and MAF > 2.5%, respectively
