# Supplementary material for: Rice diversity panel provides accurate genomic predictions for complex traits in the progenies of biparental crosses involving members of the panel
Source: Theor Appl Genet. 2017 Nov 14;131(2):417–35. doi: 10.1007/s00122-017-3011-4 (PMC5787227; doi:10.1007/s00122-017-3011-4)
Supplement: Supplementary file 6 — Supplementary material 6 (PDF 223 kb) [file 122_2017_3011_MOESM6_ESM.pdf]

Supplementary Table 6: Accuracy of genomic prediction of progeny phenotype for days to flowering (FL), nitrogen balance index (NI) and 100 panicles weight (PW), obtain with three statistical methods, Bayes B, GBLUP and RKHS, under six scenarios of relatedness between the training and the progeny set.

			FL			NI			PW		
Scenario	r^2	MAF (%)	BayesB	GBLUP	RKHS	BayesB	GBLUP	RKHS	BayesB	GBLUP	RKHS
S1	0.25	5	0.25	0.27	0.26	0.19	0.20	0.09	0.48	0.49	0.48
	0.36	5	0.28	0.30	0.28	0.24	0.24	0.13	0.50	0.52	0.49
	0.49	5	0.28	0.32	0.30	0.18	0.19	0.13	0.53	0.54	0.50
	0.64	5	0.27	0.25	0.30	0.17	0.16	0.12	0.50	0.50	0.47
	0.81	5	0.28	0.26	0.30	0.18	0.18	0.15	0.46	0.48	0.47
	0.98	5	0.27	0.24	0.28	0.20	0.19	0.23	0.45	0.49	0.45
	1a	5	0.26	0.23	0.29	0.20	0.19	0.26	0.44	0.51	0.42
	1b	2.5	0.25	0.23	0.28	0.18	0.18	0.23	0.46	0.52	0.43
S2	0.25	5	0.35	0.34	0.35	0.23	0.23	0.24	0.28	0.30	0.29
	0.36	5	0.37	0.37	0.39	0.30	0.31	0.32	0.30	0.30	0.28
	0.49	5	0.46	0.44	0.45	0.37	0.36	0.36	0.33	0.29	0.27
	0.64	5	0.40	0.41	0.42	0.40	0.38	0.39	0.31	0.31	0.28
	0.81	5	0.41	0.40	0.42	0.40	0.41	0.38	0.29	0.32	0.27
	0.98	5	0.33	0.32	0.35	0.33	0.31	0.34	0.28	0.29	0.22
	1a	5	0.31	0.27	0.31	0.24	0.22	0.27	0.31	0.33	0.28
	1b	2.5	0.31	0.28	0.32	0.23	0.22	0.28	0.36	0.37	0.34
S3	0.25	5	0.29	0.32	0.31	0.27	0.28	0.28	0.45	0.44	0.44
	0.36	5	0.33	0.32	0.29	0.34	0.36	0.36	0.46	0.43	0.43
	0.49	5	0.40	0.39	0.37	0.39	0.36	0.39	0.48	0.46	0.44
	0.64	5	0.38	0.35	0.37	0.42	0.38	0.42	0.45	0.45	0.44
	0.81	5	0.39	0.36	0.39	0.42	0.40	0.41	0.42	0.44	0.44
	0.98	5	0.35	0.32	0.34	0.38	0.40	0.38	0.38	0.41	0.40
	1a	5	0.31	0.29	0.33	0.34	0.37	0.34	0.41	0.43	0.41
	1b	2.5	0.31	0.28	0.32	0.33	0.35	0.34	0.42	0.45	0.44
	0.25	5	0.31	0.31	0.30	0.30	0.31	0.31	0.35	0.34	0.31

S4	0.36	5	0.33	0.32	0.32	0.44	0.42	0.44	0.36	0.35	0.44
	0.49	5	0.42	0.34	0.40	0.52	0.49	0.50	0.39	0.39	0.50
	0.64	5	0.45	0.38	0.43	0.48	0.47	0.48	0.39	0.39	0.48
	0.81	5	0.41	0.35	0.40	0.49	0.48	0.49	0.40	0.39	0.49
	0.98	5	0.36	0.37	0.36	0.46	0.46	0.46	0.38	0.38	0.46
	1a	5	0.31	0.35	0.30	0.45	0.45	0.45	0.37	0.38	0.45
	1b	2.5	0.27	0.31	0.28	0.42	0.42	0.43	0.38	0.39	0.43
S5	0.25	5	0.39	0.39	0.38	0.18	0.20	0.20	0.18	0.18	0.17
	0.36	5	0.40	0.39	0.38	0.34	0.32	0.34	0.21	0.19	0.19
	0.49	5	0.48	0.43	0.47	0.43	0.37	0.42	0.27	0.26	0.26
	0.64	5	0.51	0.49	0.51	0.43	0.41	0.43	0.28	0.28	0.28
	0.81	5	0.46	0.44	0.46	0.44	0.42	0.45	0.30	0.30	0.30
	0.98	5	0.39	0.43	0.40	0.41	0.40	0.41	0.29	0.29	0.29
	1a	5	0.30	0.40	0.32	0.38	0.38	0.39	0.31	0.30	0.30
	1b	2.5	0.28	0.34	0.29	0.38	0.36	0.38	0.31	0.31	0.31
S6	0.25	5	0.19 ± 0.01	0.19 ± 0.01	0.19 ± 0.01	0.12 ± 0.01	0.14 ± 0.01	0.13 ± 0.01	0.21 ± 0.01	0.24 ± 0.01	0.23 ± 0.01
	0.36	5	0.20 ± 0.01	0.19 ± 0.01	0.20 ± 0.01	0.18 ± 0.01	0.21 ± 0.01	0.20 ± 0.01	0.24 ± 0.01	0.28 ± 0.01	0.27 ± 0.01
	0.49	5	0.21 ± 0.01	0.19 ± 0.01	0.20 ± 0.01	0.22 ± 0.01	0.22 ± 0.01	0.23 ± 0.01	0.28 ± 0.01	0.33 ± 0.01	0.31 ± 0.01
	0.64	5	0.21 ± 0.01	0.22 ± 0.01	0.22 ± 0.01	0.24 ± 0.01	0.24 ± 0.01	0.26 ± 0.01	0.30 ± 0.01	0.35 ± 0.01	0.33 ± 0.01
	0.81	5	0.19 ± 0.01	0.19 ± 0.01	0.18 ± 0.01	0.24 ± 0.01	0.23 ± 0.01	0.26 ± 0.01	0.32 ± 0.01	0.36 ± 0.01	0.35 ± 0.01
	0.98	5	0.12 ± 0.01	0.12 ± 0.01	0.11 ± 0.01	0.21 ± 0.02	0.21 ± 0.02	0.24 ± 0.01	0.30 ± 0.01	0.34 ± 0.01	0.33 ± 0.01
	1a	5	0.06 ± 0.01	0.07 ± 0.01	0.06 ± 0.01	0.20 ± 0.02	0.18 ± 0.02	0.23 ± 0.02	0.30 ± 0.01	0.34 ± 0.01	0.33 ± 0.01
	1b	2.5	0.06 ± 0.01	0.07 ± 0.01	0.05 ± 0.01	0.19 ± 0.02	0.19 ± 0.02	0.23 ± 0.02	0.29 ± 0.01	0.33 ± 0.01	0.32 ± 0.01

1-a and 1-b, represent incidence matrixes without selection on r^2, but filtered with MAF > 5 % and MAF > 2.5%, respectively
