# Supplementary figures and images for: Rice diversity panel provides accurate genomic predictions for complex traits in the progenies of biparental crosses involving members of the panel
Source: Theor Appl Genet. 2017 Nov 14;131(2):417–35. doi: 10.1007/s00122-017-3011-4 (PMC5787227; doi:10.1007/s00122-017-3011-4)

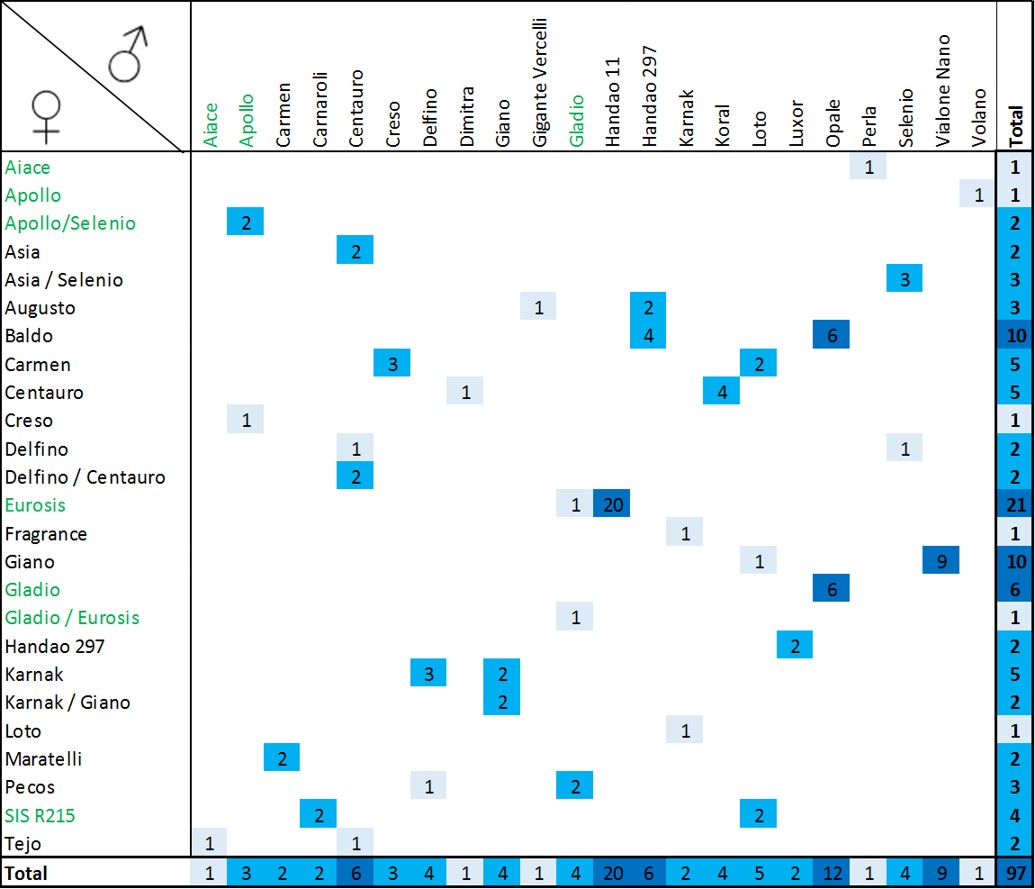

Supplement: Supplementary file 7 — Supplementary material 7 (JPEG 124 kb) [file 122_2017_3011_MOESM7_ESM.jpg]

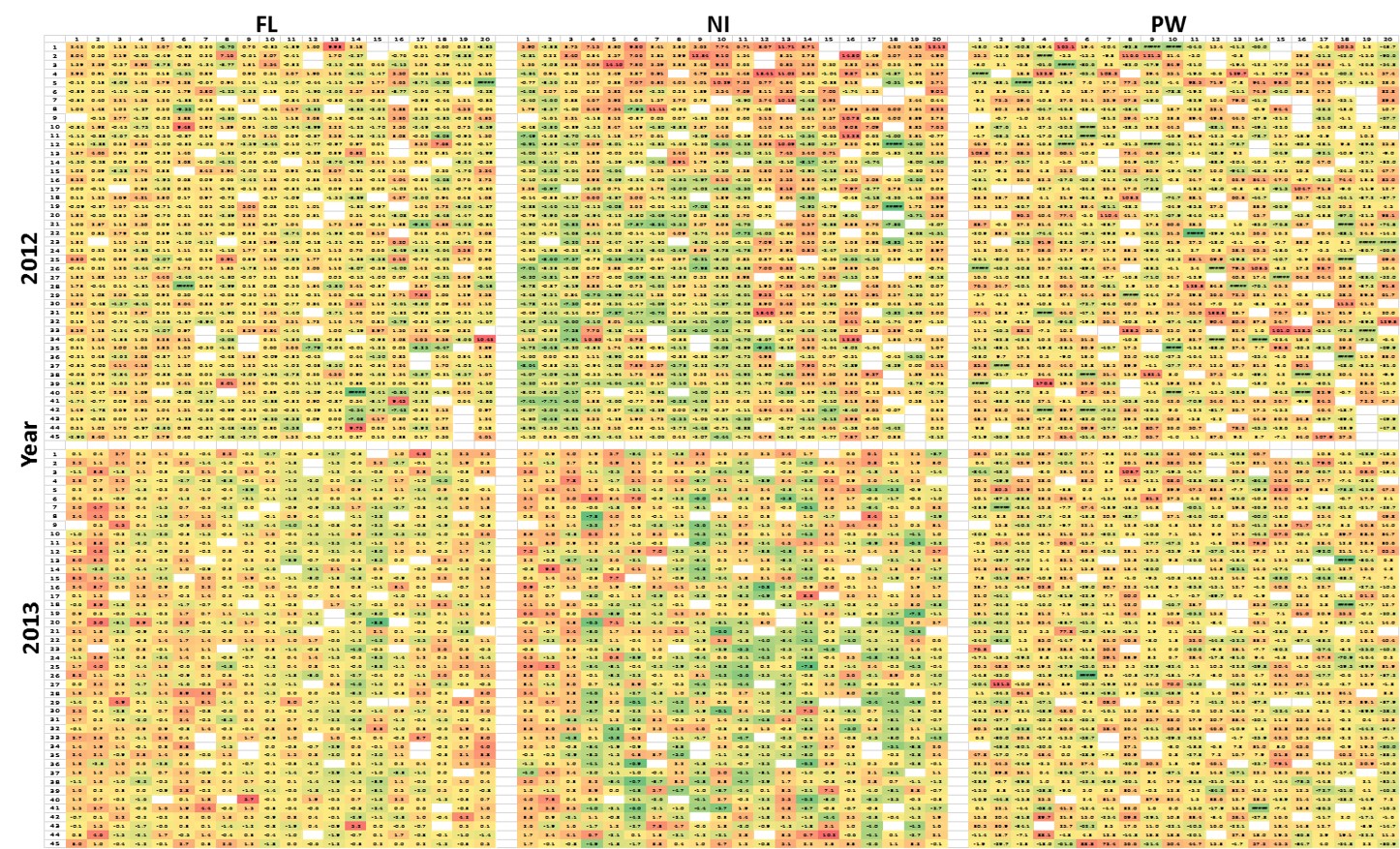

Supplement: Supplementary file 8 — Supplementary material 8 (JPEG 692 kb) [file 122_2017_3011_MOESM8_ESM.jpg]

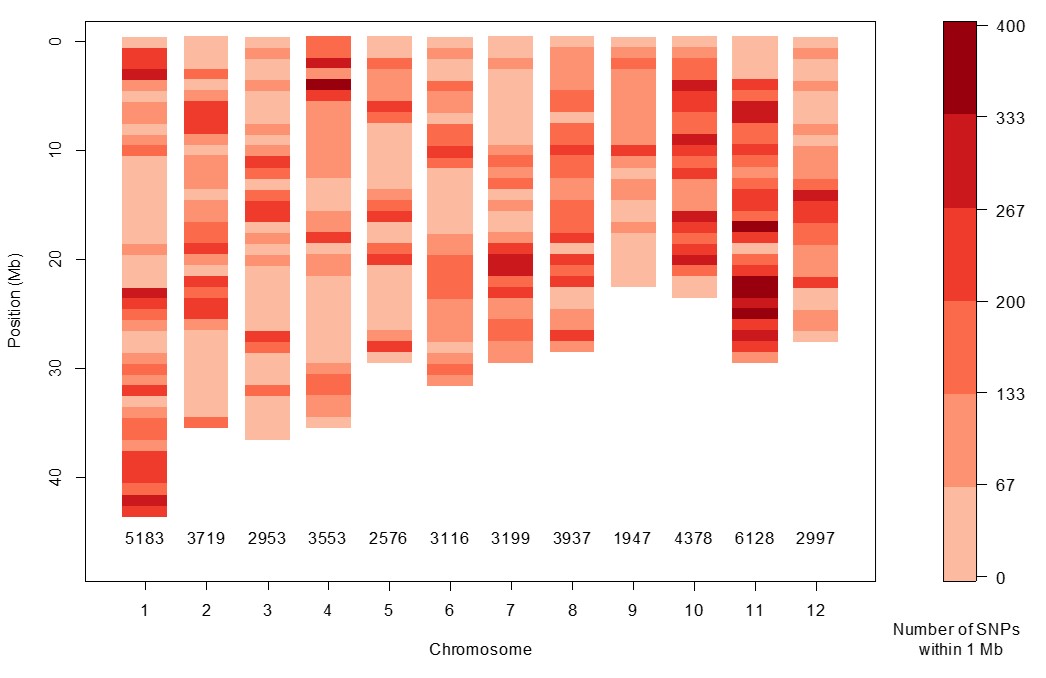

Supplement: Supplementary file 9 — Supplementary material 9 (JPEG 85 kb) [file 122_2017_3011_MOESM9_ESM.jpg]

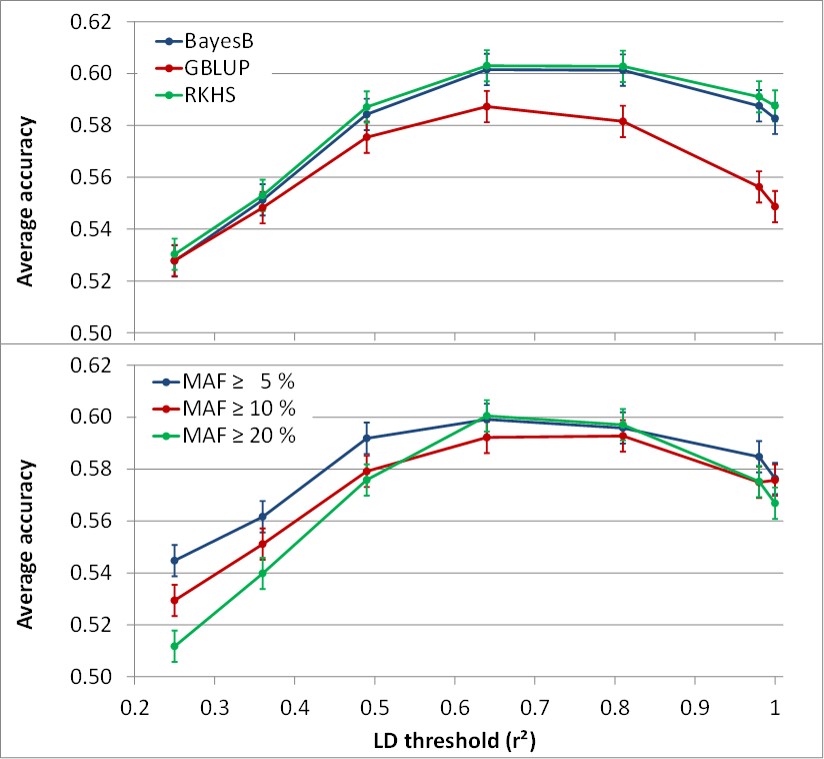

Supplement: Supplementary file 10 — Supplementary material 10 (JPEG 98 kb) [file 122_2017_3011_MOESM10_ESM.jpg]

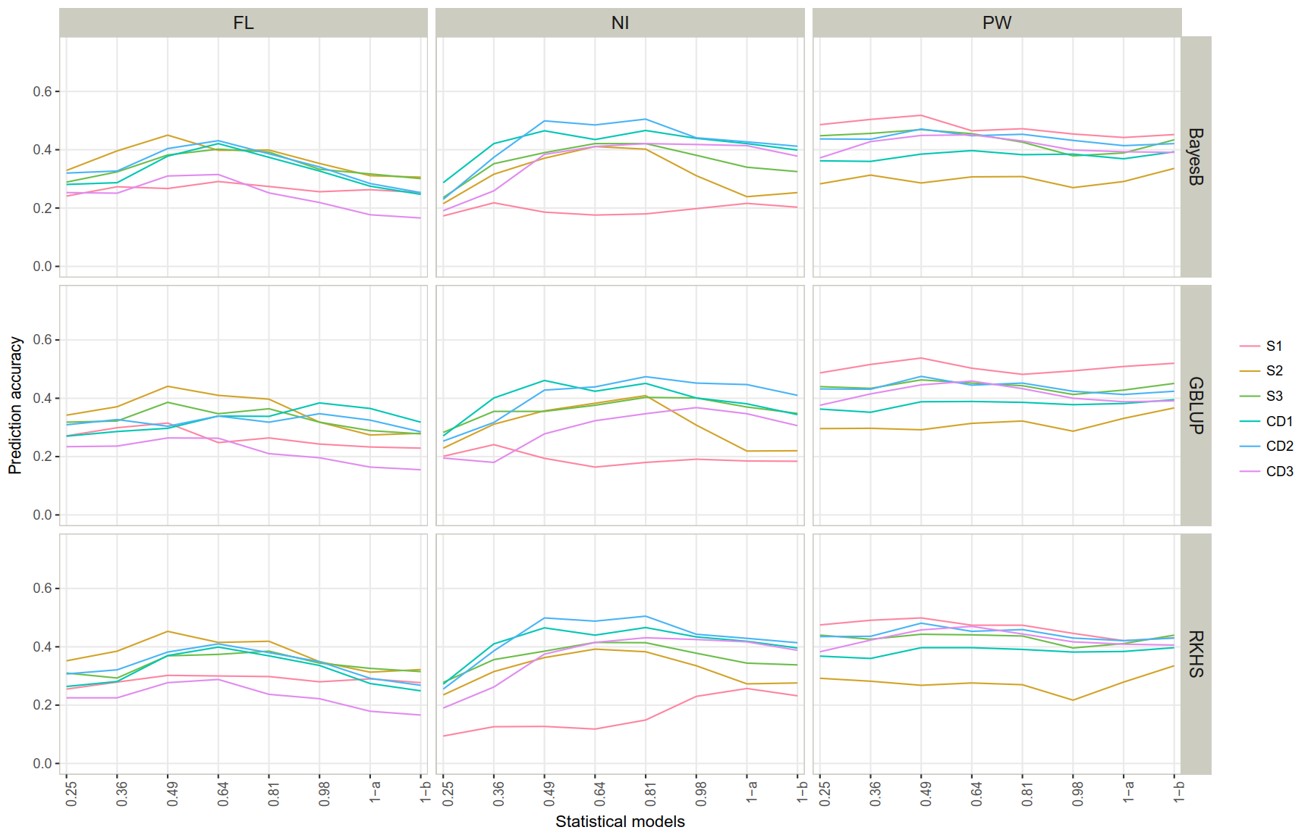

Supplement: Supplementary file 11 — Supplementary material 11 (JPEG 156 kb) [file 122_2017_3011_MOESM11_ESM.jpg]

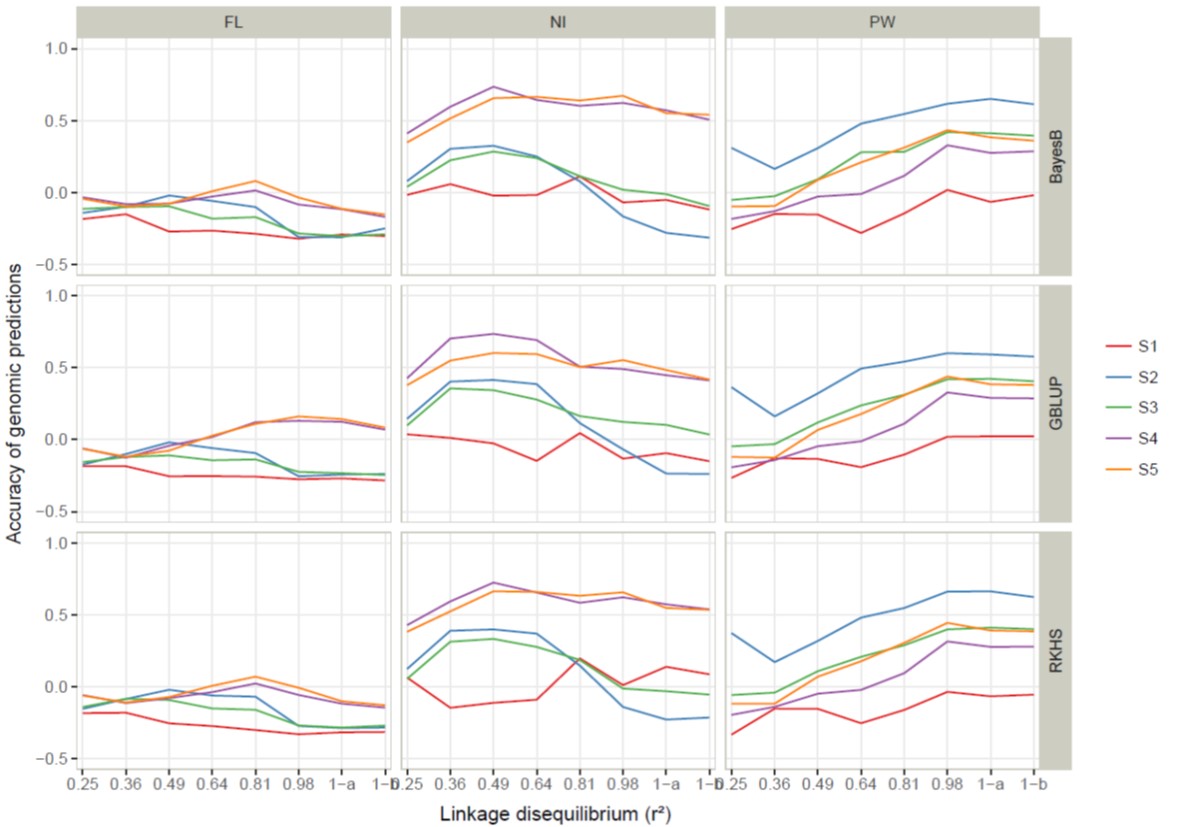

Supplement: Supplementary file 12 — Supplementary material 12 (JPEG 138 kb) [file 122_2017_3011_MOESM12_ESM.jpg]

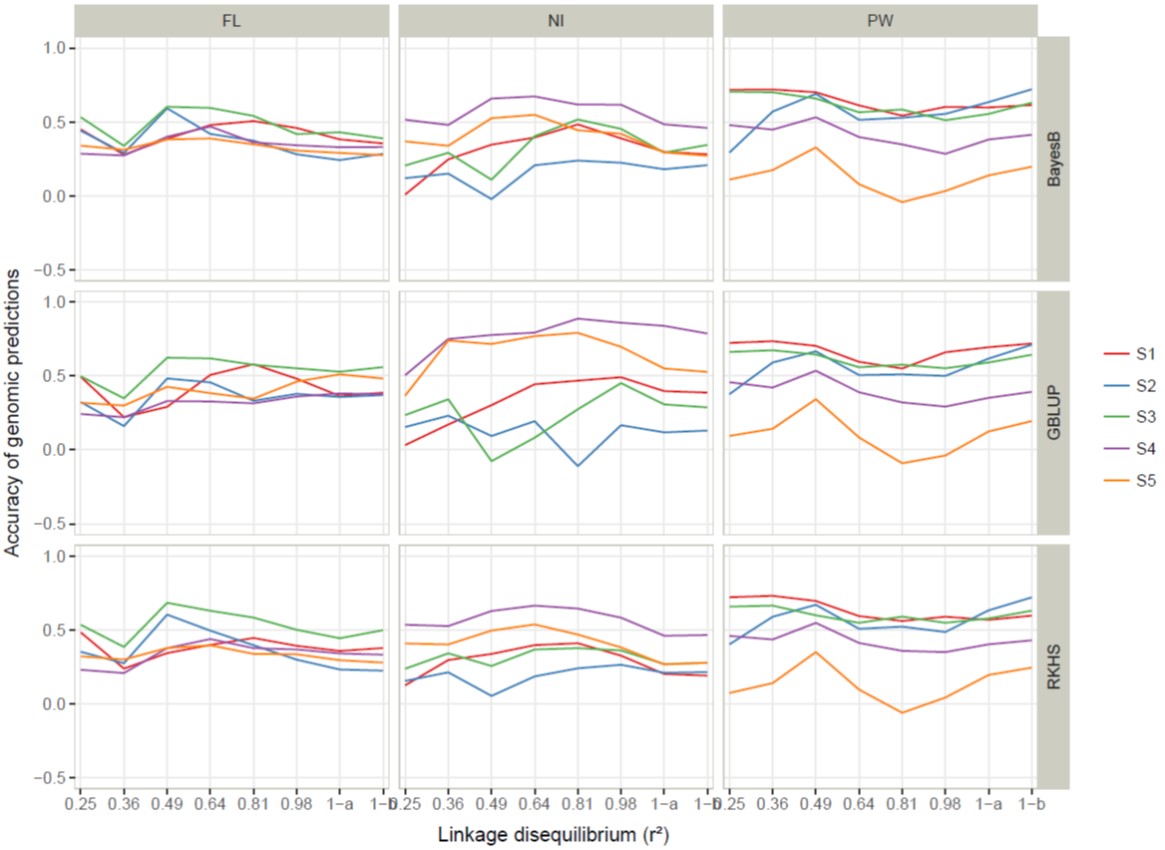

Supplement: Supplementary file 13 — Supplementary material 13 (JPEG 141 kb) [file 122_2017_3011_MOESM13_ESM.jpg]

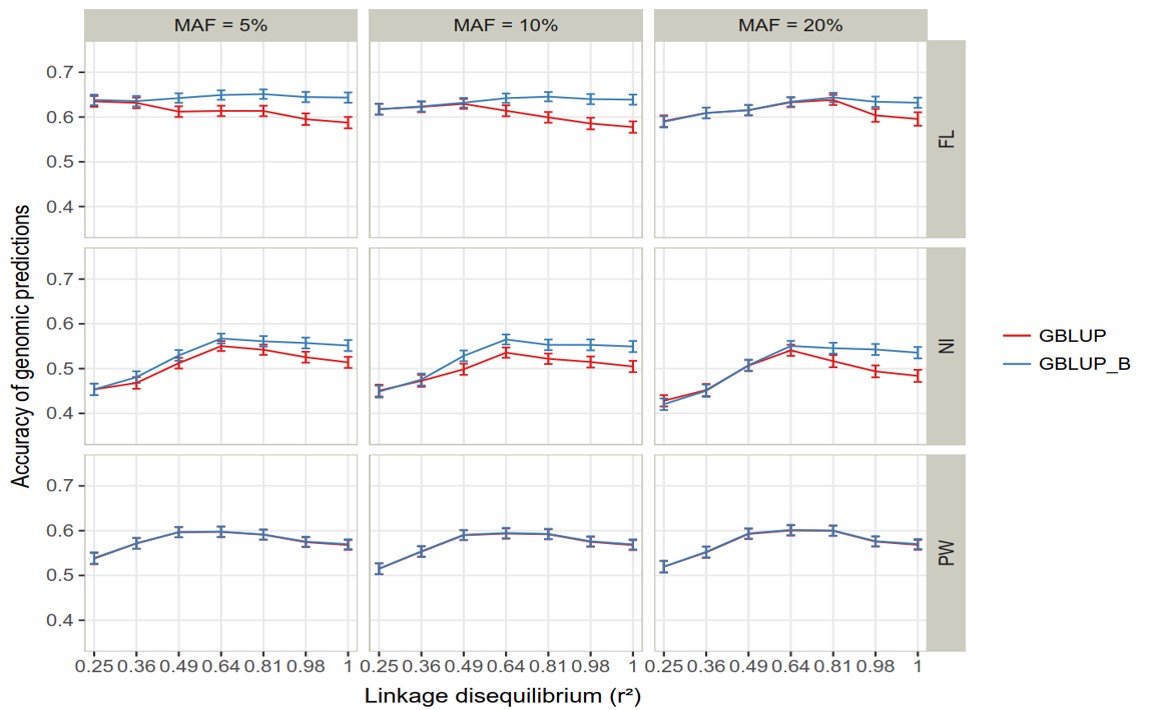

Supplement: Supplementary file 14 — Supplementary material 14 (JPEG 116 kb) [file 122_2017_3011_MOESM14_ESM.jpg]
